# Phenotypic coupling of sleep and starvation resistance evolves in *D. melanogaster*

**DOI:** 10.1186/s12862-020-01691-8

**Published:** 2020-09-22

**Authors:** Didem P. Sarikaya, Julie Cridland, Adam Tarakji, Hayley Sheehy, Sophia Davis, Ashley Kochummen, Ryan Hatmaker, Nossin Khan, Joanna Chiu, David J. Begun

**Affiliations:** 1 Department of Evolution and Ecology, University of California Davis, Davis, California USA; 2 Department of Molecular and Cellular Biology, University of California Davis, Davis, California USA; 3 Department of Nematology and Entomology, University of California Davis, Davis, California USA

**Keywords:** Local adaptation, Sleep, Starvation resistance, Behavior, Trade-off, Plasticity

## Abstract

**Background:**

One hypothesis for the function of sleep is that it serves as a mechanism to conserve energy. Recent studies have suggested that increased sleep can be an adaptive mechanism to improve survival under food deprivation in *Drosophila melanogaster*. To test the generality of this hypothesis, we compared sleep and its plastic response to starvation in a temperate and tropical population of *Drosophila melanogaster*.

**Results:**

We found that flies from the temperate population were more starvation resistant, and hypothesized that they would engage in behaviors that are considered to conserve energy, including increased sleep and reduced movement. Surprisingly, temperate flies slept less and moved more when they were awake compared to tropical flies, both under fed and starved conditions, therefore sleep did not correlate with population-level differences in starvation resistance. In contrast, total sleep and percent change in sleep when starved were strongly positively correlated with starvation resistance within the tropical population, but not within the temperate population. Thus, we observe unexpectedly complex relationships between starvation and sleep that vary both within and across populations. These observations falsify the simple hypothesis of a straightforward relationship between sleep and energy conservation. We also tested the hypothesis that starvation is correlated with metabolic phenotypes by investigating stored lipid and carbohydrate levels, and found that stored metabolites partially contributed towards variation starvation resistance.

**Conclusions:**

Our findings demonstrate that the function of sleep under starvation can rapidly evolve on short timescales and raise new questions about the physiological correlates of sleep and the extent to which variation in sleep is shaped by natural selection.

## Background

Sleep is an ancestral animal behavior [1–3]. The ancient nature of the trait demonstrates its biological importance, yet the functions of sleep remain contentious. It is connected to memory consolidation [4, 5], energy conservation [6, 7], and reducing exposure to hazards during periods where foraging is not advantageous [8]. While sleep itself is ancient, it nevertheless evolves. For example, duration of sleep is highly variable across different species, and may evolve in response to foraging demands [9], predation risk [10], and variation in immune states [11].

Sleep plasticity allows animals to regulate their sleep in response to environmental variation. In birds, drastic reductions in sleep have been observed during mating season [12], under increased predation risk [13] and during migration [14]. These studies did not note a reduction in performance for animals sleeping less, suggesting that animals can, at least in some cases, attenuate sleep without incurring negative fitness effects. In contrast, food deprivation commonly reduces sleep across animals [15–17], and multiple studies have found a trade-off between energy expending behavior (wakefulness and foraging) and length of survival under food deprivation [18, 19]. Therefore, there are multiple instances where suppression of sleep has no obvious fitness effect, and other instances where it does.

In instances where suppression of sleep has a fitness effect, it is considered to be a result of sleep functioning to conserve energy [7]. This has been explored most in *D. melanogaster* where an emerging hypothesis posits that increased sleep regulates starvation resistance by conserving energy. This hypothesis has been supported by studies on one Japanese population [20], on inbred lines from Raleigh [21], and on a collection of lines from around the world [19]. Similarly, laboratory selection of *D. melanogaster* for starvation resistance leads to increased sleep [18, 22]. There may be a trade-off between sleep-wake activity and starvation resistance, as animals that sleep less would likely deplete stored resources faster, thus reducing life span when deprived of food. However, these studies either sampled a single population [20, 21] or only used one line to represent a population from around the world [19]. Therefore, a population-level comparison of sleep and starvation resistance, which is necessary to reveal the mechanisms of evolutionary divergence, has not been conducted to date. Moreover, whether sleep under starvation functions as an energy conservation mechanism broadly in *D. melanogaster* remains unclear.

Latitudinal clines in the *D. melanogaster* model system provide an excellent substrate for investigating the genetic and phenotypic mechanisms of population divergence. These clines have been independently established on multiple continents and often show parallel patterns of phenotypic and genetic differentiation [23–27]. Detailed analysis of phenotypes in clinal populations from North America and Australia support the idea that several phenotypes, including key life history traits, have diverged between high and low latitude populations as a result (direct or indirect) of selection (reviewed in Hoffmann and Weeks 2007, Adrion et al. 2015). As a first step in investigating the possible effects of selection on the connection between behavior and starvation resistance, we studied two populations of *D. melanogaster* from the “endpoints” of the well-studied American cline, one temperate (Maine USA) and the other tropical (Panama City, Panama). Previous studies sampling along the cline from Maine to Florida or sampling the endpoints have both shown that the high latitude populations are more starvation resistant [28, 29]. Therefore we hypothesized that the temperate population would be more starvation resistant compared to the tropical population, and that this starvation resistance would correlate with increased sleep and reduced movement under starvation to conserve energy. However, we did not find support for sleep serving an energy conservation function across the two populations, falsifying this hypothesis at the population-level. In contrast, within-population differences in starvation resistance were positively correlated with sleep in the tropical population, but not in the temperate population. Our results highlight the complexity of divergence of behavioral and physiological traits in locally adapted populations, and suggest that the connection between sleep and starvation may evolve on very short timescales.

## Results

To test whether starvation resistance evolves through changes in sleep, we quantified behavioral traits in 10 isofemale lines each from two populations sampled from endpoints of the well-studied North American cline, Maine (ME) and Panama City (PC). Based on previous work [19, 28, 29], we hypothesized that they would be more starvation resistant, and behavioral patterns that conserve energy, such as increased sleep and reduced locomotion under starvation, would contribute to their increased starvation resistance compared to the PC population.

**Fig. 1:**
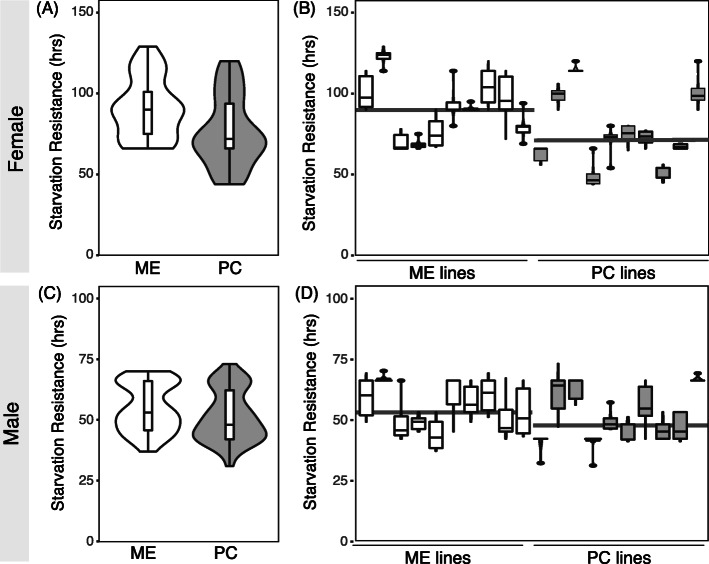
ME flies are more starvation resistant compared to PC. **a,** ) Hour where half of the flies died of starvation for ME (white) and PC (gray) in (**a**) females and (**c**) males reared and maintained at 25 °C. Plot contains all data points from the 10 lines per population. **b, d** Boxplot of starvation resistance in hours for individual lines for ME and PC (**b**) females and (**d**) males. Solid line across the plot denotes the average value for each population

First, we compared starvation resistance in both populations and found that ME flies were more starvation resistant than PC flies for both sexes (Fig. 1a, c; Supplemental Table 1; t-test *p* < 0.0001, ANOVA *df* = 1, *F* = 88.63, *p* < 0.0001). Females were more starvation resistant than males for both populations, and the population-level differences were more striking in females as well. Comparing starvation resistance in the 10 lines per population, we found considerable differences in starvation resistance within ME and PC populations (Fig. 1b, d; ANOVA *df* = 18, *F* = 18.23, *p* < 0.0001). Males and females of each line showed very similar patterns, suggesting that genetics drives starvation resistance and that these factors are not sex-specific. These results highlight that the ME population has evolved higher starvation resistance, but the trait remains variable within each population.

### ME flies sleep less and move more than PC flies when fed

**Fig. 2:**
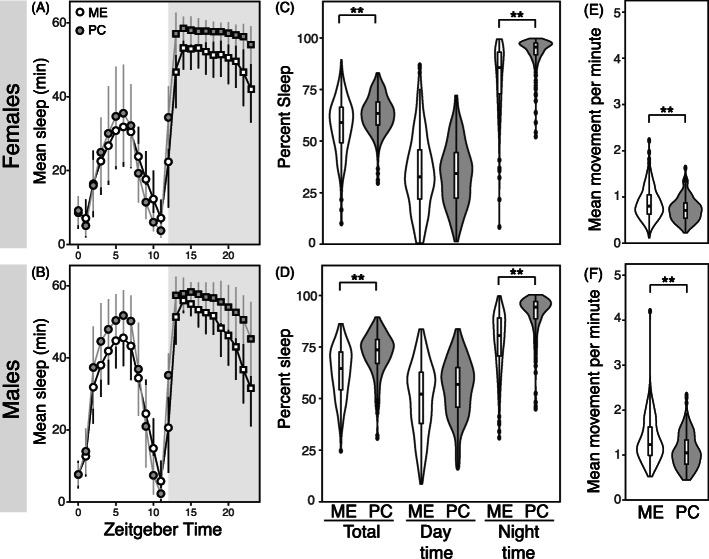
ME flies sleep less at night and move more when awake compared to PC. **a-b** Mean sleep and standard error by Zeitgeber hour of (**a**) females and (**b**) males from ME (white) and PC (gray) populations at 25 °C during 12 h of light and 12 h dark periods, where night time is indicated by the gray shading of the plot. **c-d** Percentage of total sleep over a 24 h day (left), light period (middle) and night time (right) in ME and PC (**c**) females and (**d**) males. PC results are shaded in gray, and asterisks denote statistical significance after Bonferroni correction. Data points for daytime are denoted in circles and nighttime are in squares. **e-f** Average locomotor activity per minute when awake of (**e**) females and (**f**) males of ME and PC populations at 21 °C and 25 °C. Asterisks denote statistical significance after Bonferroni correction

To test whether sleep contributes to differences in starvation resistance, we measured sleep under well-fed conditions in ME and PC flies. Total sleep was calculated as the percentage of time in a 24 h day that the flies spent sleeping. Total sleep was higher in PC compared to ME (Fig. 2a-d; Supplemental Table 2; t-test *p* < 0.0001, ANOVA *df* = 1, *F* = 229.73, *p* < 0.0001) for both sexes. Day sleep was similar for both populations (Fig. 1C-D; t-test *p* = 0.81; ANOVA *df* = 1, *F* = 5.11, *p* = 0.02), but PC flies consistently slept more at night compared to ME (Fig. 1c-d; t-test *p* < 0.0001; ANOVA *df* = 1, *F* = 71,401, *p* < 0.0001). *Drosophila* sleep is polyphasic, with number and length of sleep bouts determining total sleep. Bout number was significantly higher in ME, while bout length was significantly longer in PC (Supplemental Table 2; t-test *p* < 0.0001; ANOVA bout number *df* = 1, *F* = 132.25, *p* < 0.0001, bout length *df* = 1, *F* = 185.51, *p* < 0.0001). Overall, we found that the population differences in sleep between ME and PC flies primarily resulted from differences in night time sleep through increased bout length in PC flies.

We next compared locomotor activity of flies, as measured by the number of times flies crossed the infrared beam of DAMS when awake. Higher levels of activity can indicate higher metabolic rate in various species, including *D. melanogaster* [30]. ME flies had higher average movement per minute when awake for both sexes, suggesting that they are more active than PC flies (Fig. 1e-f; Supplemental Table 2; t-test *p* < 0.0001; ANOVA *df* = 1, *F* = 107.49, *p* < 0.0001). Taken together, we found that ME flies engage in more energetically costly behavior compared to PC flies under well-fed conditions.

### ME flies engage in energetically costly behaviors when starved

The higher locomotor activity and lower sleep in the ME population conflicted with our initial hypothesis that starvation resistance would evolve through increased sleep and reduced movement, based on previous work conducted in *D. melanogaster* [19, 20, 22]. We therefore hypothesized that ME flies may sleep more when starved compared to the PC flies.

**Fig. 3:**
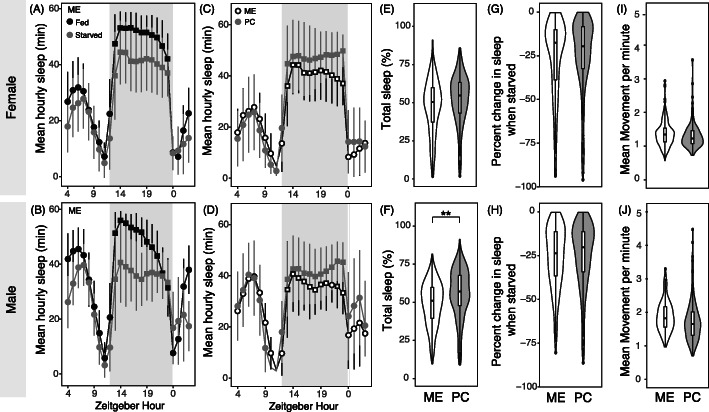
PC flies sleep more and engage in fewer locomotor activity when awake than ME under starvation. **a-b** Comparison of sleep patterns in ME flies when fed (black) and starved (gray). Data points for daytime are denoted in circles and nighttime are in squares. **c-d** Comparison of hourly sleep patterns of ME (white) and PC (gray) during starvation. Gray box denotes dark period, and Zeitgeber Hour denoted in these graphs start with when flies were placed on starvation media. **e-f** Total percent sleep when starved for ME and PC females and males. **g-h** Percent change in sleep upon starvation at the individual level for ME and PC flies. **i-j** Mean movement per minute when awake under starvation for (**i**) females and (**j**) males. Asterisks denote statistical significance after Bonferroni correction

We observed that starvation reduced sleep for both sexes and populations (Fig. 3a-b; Supplemental Table 3; t-test *p* < 0.0001). The decrease in sleep was caused by decreases in bout length (t-test *p* < 0.0001), while bout number remained unaffected by starvation (Supplemental Table 3; t-test *p* > 0.05). A previous study showed that males of inbred laboratory lines of *D. melanogaster* do not show behavioral differences during the first 12 h of starvation, regardless of whether starvation was induced during the day or night [16]. In contrast, we found that starved males slept less, even in the first few hours after starvation (Supplemental Figure 1). Given that we did not see a delay in behavioral change in starved males, we compared both females and males in subsequent analyses.

Even under starvation, PC males slept more than ME males (Fig. 3f; Supplemental Table 2; t-test *p* < 0.0001. Though differences were not significant, a similar trend was observed for females (Fig. 3e; Supplemental Table 2; t-test *p* = 0.005, not significant after Bonferroni correction). Similar to when flies were fed, bout length was longer and bout number was lower in starved PC flies compared to starved ME flies (Supplemental Table 2; t-test *p* < 0.0001). To test whether the proportional effect of starvation on sleep differs between populations, we calculated the percent change in sleep of each individual pre- vs. post-starvation. We observed no population differences across sexes (Fig. 3g-h; Supplemental Table 2; t-test *p* > 0.05). When we pooled data from both populations, we observed a weak positive correlation between percent change in sleep and starvation resistance in females at 25 °C (*R*^*2*^ = 0.24, *p* < 0.05), but did not observe any correlation in other conditions (Supplemental Figure 2; *R*^*2*^ < 0.15).

**Fig. 4:**
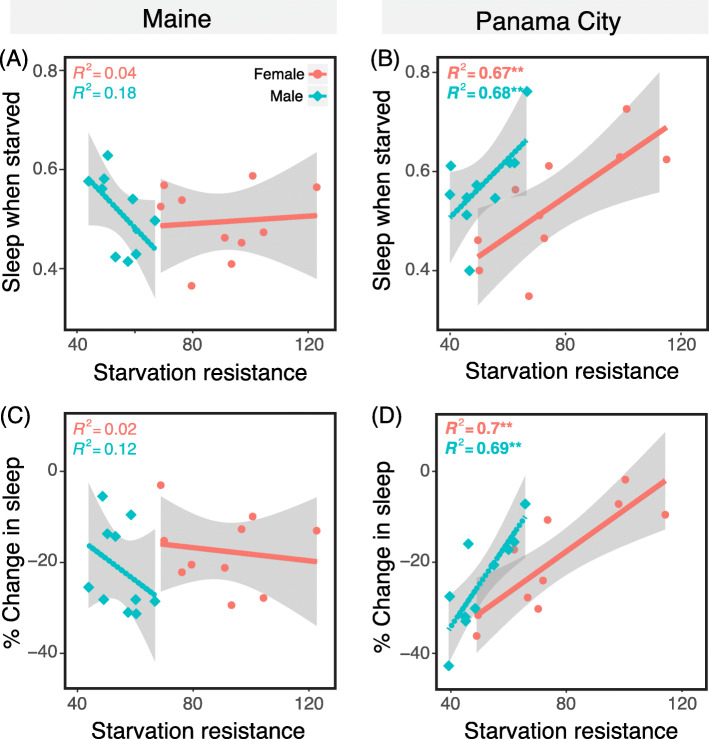
Coupling of starvation resistance and sleep is different in ME and PC populations. **a-b** Regression analysis of starvation resistance and percent of the 24 h day flies slept when starved for (**a**) ME and (**b**) PC. **c-d** Regression analysis of starvation resistance and percent change in sleep when starved for (**c**) ME and (**d**) PC flies. Each data point denotes the average of one of the 10 lines. Female data points are red circles with a solid regression line, while males are blue diamonds with a dashed line. The grey area denotes the confidence interval. * denotes *p* < 0.05 and ** denotes *p* < 0.01

Lastly, we compared activity patterns of fed and starved animals. Starvation led to higher activity for both populations and sexes (Supplemental Table 2; t-test *p* < 0.001). Under starvation, ME flies moved more when awake than PC flies, though population differences were not significant (Fig. 4i-j; Supplemental Table 2; t-test *p* = 0.026 for female and *p* = 0.003 for male). Thus, the ME population showed reduced sleep and increased activity relative to PC and similar levels of suppression of sleep when starved, despite being more starvation resistant.

### Coupling of sleep and starvation resistance is an evolvable trait

A previous study showed that starvation resistance correlates with sleep within one Japanese population [20]. While sleep patterns did not explain the population-level difference in starvation resistance, we asked whether sleep may explain within-population differences in starvation resistance that we observed earlier. When we tested the regression of the total percentage of sleep when starved and percent change in sleep vs. starvation resistance, we found a striking population difference in correlations. Sleep-when-starved and percent-change-in-sleep-when-starved were strongly positively correlated with starvation resistance in PC flies (Fig. 4c-d). In contrast, ME flies showed no relationship between these factors (Fig. 4a-b). These results demonstrate that the plastic sleep response to starvation has diverged between these populations, and that energetically costly behaviors (wakefulness) can be coupled and decoupled from starvation within species.

### Metabolic differences contribute towards population-level differences in starvation resistance

Along with behavioral differences, another major contributor for evolution of starvation resistance is stored metabolites. Studies from natural populations and laboratory selection lines for increased starvation resistance show that increased stored lipid levels can lead to higher starvation resistance [19, 22, 31], and a previous study has shown increased lipid storage in a population from Vermont compared to Florida [32]. In addition, stored glucose levels correlate with percent change in sleep when starved in females [19]. Therefore we hypothesized that stored metabolites may explain part of the population-level differences in starvation resistance that we observed.

**Fig. 5:**
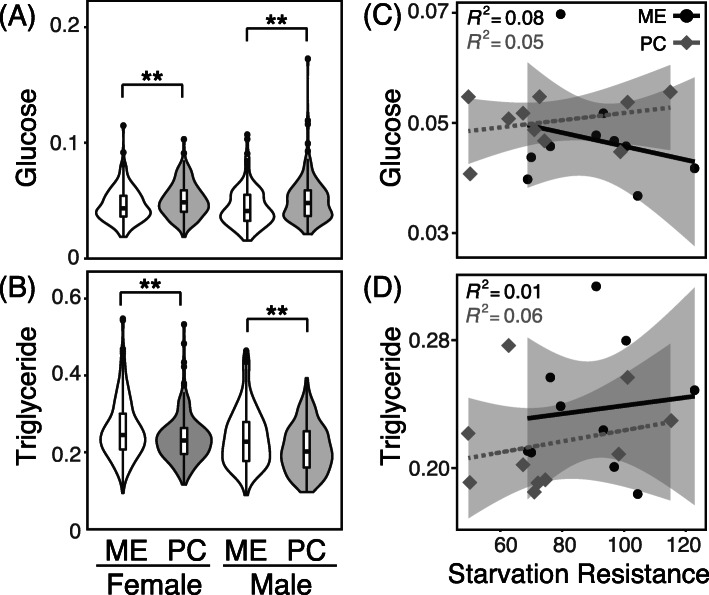
Stored metabolites are different in ME and PC populations. **a** Whole body glucose (mg glucose / mg protein) and (**b**) TGA (mg TGA / mg protein) in ME (while) and PC (gray) female and males. **c-d** Regression plots of starvation resistance and (**c**) glucose or (**d**) TGA levels in females. Black circles and solid lines denote the ME population and grey diamonds and dashed lines denote the PC population. The grey shaded area represents the confidence intervals of the regression. Panels **a-b** present distributions from each population / sex combination, while points in panel **c** and **d** represent the line means for each population. * denotes *p* < 0.05 and ** denotes *p* < 0.01

Using enzymatic kits, we measured whole body glucose and triglyceride (TGA) levels. Whole body glucose was significantly higher in PC than ME (Fig. 5a; Supplemental Table 1; t-test *p* < 0.005). Whole body TGA levels were significantly higher in ME compared to PC (Fig. 5b; Supplemental Table 1; t-test *p* < 0.0001). We observed no effect of sex on glucose or TGA levels. Regression analyses for the effects of stored lipid or sugar levels did not reveal strong patterns with starvation resistance or percent change in sleep (Fig. 5c-d).

### Temperature affects metabolism and sex-specific patterns in sleep

Temperature influences sleep across most animals, and most attributes of *D. melanogaster* biology can be affected by temperature [33]. All of the experiments described above were performed on flies reared and maintained at 25 °C, the most common experimental condition for *D. melanogaster*. To test whether temperature may affect sleep, metabolism and their role in starvation resistance, we tested these components at 21 °C.

Population-level differences in starvation resistance was more pronounced at 21 °C (Supplemental Table 1; t-test *p* < 0.0001). We also found that glucose levels were significantly affected by temperature, and both ME and PC populations showed a significant increase in glucose levels at 21 °C (Supplemental Table 1; t-test *p* < 0.001). Overall, our behavioral results were highly similar at 25 °C and 21 °C, with the exception of the behavior of PC females at 21 °C. PC females displayed a significant reduction in bout length and increase in bout number at 21 °C compared to 25 °C, and this temperature effect was not observed in PC males (Supplemental Table 4; t-test *p* < 0.0001 for female, *p* = 0.49 for male). The significant increase in bout number was also observed in PC females under starvation at 21 °C. In addition, we observed a consistent increase in night time bout length at 21 °C in males of both populations (Supplemental Table 4; t-test *p* < 0.0001). These results highlight that sex-specific behavioral shifts to temperature can evolve in different populations.

### Variation in starvation resistance correlates with metabolic and behavioral traits

To further understand the effects of behavioral and metabolic traits on variation in starvation resistance, we used linear and multilevel Bayesian models (Table 1, Supplemental Table 5). We separated the analyses to include either metabolism or behavioral traits during starvation. The QR decomposition of both models found that our models had enough degrees of freedom to estimate the number of variables used (Supplemental File 1).

**Table 1:** Multilevel Bayesian model of starvation resistance. Summary of multilevel Bayesian model of starvation resistance incorporating environmental and metabolic parameters (top) and behavioral parameters (bottom). Effect represents the mean of the posterior distribution for the parameters in the model. For example, the estimate of LD50 (starvation resistance) is reduced for males relative to females for both metabolism and behavior. Similarly, the Panama City population (PC) has reduced starvation resistance relative to Maine. Est. Error is the estimate of the error; l-95% and u-95% are the lower and upper credible intervals

	Starvation resistance			
	Estimate	Est. Error	1–95% CI	u-95% CU
Sex (M)	−9.38	2.74	− 14.71	−3.92
Temperature (25 °C)	−7.99	2.7	− 13.23	−2.68
Population (PC)	− 4.06	2.53	− 8.95	0.95
Glucose (mg Glu / mg Protein)	3.95	2.46	−0.92	8.79
Triglyceride (mg TGA / mg Protein)	0.42	2.97	−5.38	6.2
Sex (M)	−8.71	2.73	−14.03	−3.35
Temperature (25 °C)	−8.8	2.63	−13.8	−3.5
Population (PC)	−4.46	2.51	−9.32	0.47
Percent change in sleep	0.54	0.19	0.17	0.93
Starved mean movement (beam breaks / min)	−2.98	2.83	−8.49	2.54
Total sleep when starved	−0.02	2.98	−5.77	5.83

As expected, we observed a strong effect of population, sex and temperature for all analyses (Table 1, Supplemental Table 5). Interestingly, metabolic or behavioral traits that contributed towards the variation of starvation resistance were inconsistent between the linear and Bayesian analysis. The linear model found a weak effect of TGA on starvation resistance (*t* = 2.41, *p* = 0.018), while the Bayesian model found a weak effect of glucose. Total sleep under starvation significantly contributed towards variation in starvation resistance under the linear model (Supplemental Table 5; *t* = 3.9, *p* = 0.0002), while the variables of percent change in sleep and mean movement when starved showed correlations with starvation resistance in the Bayesian model (Table 1). The linear models for metabolism and behavior had adjusted R-squared values of 0.71 and 0.76, respectively. The Bayesian model’s rHat value converged to 1 and the density plot of the observed results overlapped with predicted results for both analyses (see Supplemental File 1), indicating that the fit of the model was high. These results suggest that both metabolic and behavioral traits contribute towards variation in starvation resistance and that traits can affect starvation resistance in contrasting ways.

## Discussion

Here, we tested the hypothesis that sleep functions to conserve energy during starvation and that divergence in sleep patterns contributes to population-level differences in starvation resistance. However, our results falsified a simple hypothesis for sleep’s function in energy conservation. In general, ME flies were more starvation resistant, slept less and in a more fragmented manner, and moved more when awake. Therefore, our results do not support the hypothesis that sleep is integral to the evolution of starvation resistance in flies. However, we found that increased sleep was indeed correlated with higher starvation resistance in the PC population, highlighting that the relationship between sleep and starvation resistance, and by proxy the function for sleep under starvation, can evolve rapidly within a species.

We found that the ME population was more starvation resistant compared to the PC population, which is consistent with previous studies that found increased starvation resistance in high latitude populations of the North American cline [28, 29]. However, the relationship between latitude and starvation resistance varies across continents, as previous studies have identified a weak cline or no cline in Australia [34, 35], a negative correlation between starvation resistance and latitude in India [31, 36], and a negative or no correlation in South America [37, 38]. The strong seasonal variability in the environment of the East coast of North America may lead to distinct selective pressures in the temperate regions that are different from the Australian, Indian and South American populations that have been sampled to date. Sleep has not been studied in these populations, therefore it remains unclear whether the correlation with decreased sleep in high latitude populations is consistent across continents.

We were particularly intrigued by the falsification of our hypothesis when comparing the two populations, but support for our hypothesis within the PC population. Studies that supported the idea that increased sleep is an adaptive mechanism to increase starvation resistance were conducted on laboratory selection lines [18, 22], a selection of single lines that originated from across the world [19], and lines from a single population [20, 21]. Laboratory selection studies can illuminate genetic pathways and mechanisms that may underlie the evolution of traits, yet studies of natural populations highlight that the same phenotypic outcome can be achieved through alternative mechanisms. For example, laboratory selection for increased starvation resistance consistently leads to increased lipid storage in flies [18, 39], yet natural populations have evolved starvation resistance without changes in lipid levels [38]. Similarly, examining lines from around the world can highlight general patterns that exist within species, but will not capture the complexities that exist in each population. When comparing results, the PC population findings are similar to Miura et al. (2019), and the ME population showed a novel pattern where reduced sleep did not have negative effects on survival, similar to patterns observed in birds [12–14]. These findings suggest that population-level analyses are important when further exploring the evolution of sleep in *Drosophila*.

Studies focusing on the evolution of starvation resistance in *Drosophila* in the last three decades have found varying contributions of stored metabolites to starvation resistance. As mentioned above, laboratory selection for starvation resistance appears to consistently correlate with increased lipid storage [18], but lipid levels in natural populations of *Drosophila* do not always correlate with starvation resistance [31, 38]. Genetic panels and sampling of lines from various populations does reveal a positive correlation between lipids and starvation resistance [19, 40], highlighting that there may be general phenotypic correlations between lipid storage and starvation resistance in *D. melanogaster*, but starvation resistance can evolve through other mechanisms. Here, we found increased TGA levels and decreased glucose levels in the starvation resistant ME population. Linear and Bayesian models for starvation resistance found a contribution of TGA and glucose, respectively, and stored lipid or carbohydrate levels may contribute towards the evolution of starvation resistance in these populations. This is consistent with the idea that many small effects can contribute to a trait and that larger experiments are needed to detect effects of smaller size. A recent study found that flies carrying a natural genetic variant of *foxo* are starvation resistant through increased catabolization of fat [41]. Similarly, the ME population may be more efficient at catabolism than the PC population, thereby surviving for longer periods of time under food deprivation.

Temperature is one of the main environmental factors inducing phenotypic plasticity of sleep. For example, warmer temperatures are associated with decreased sleep in birds [42], temperatures above and below 25 °C reduces sleep in humans [43], and exposure to temperatures above 29 °C are associated with increased daytime sleep and decreased night time sleep in flies [44, 45]. To our knowledge, the sex-specific differences in temperature-induced plasticity of sleep has not been demonstrated. We observed effects on sleep parameters from only a 4 °C temperature change and these effects were sex-specific. Males had shorter sleep bouts at 25 °C compared to 21 °C, similar to a previous report [44], while their total sleep duration was unaffected. In contrast, sleep in ME females was unaffected by temperature, while PC females had longer and fewer sleep bouts at 25 °C. All females used in our experiments were kept with males, therefore were likely mated. Sex peptides transferred from males dramatically alter female sleep behavior, and these sex-specific differences in temperature response may be caused by evolution in sex peptide genes [46, 47]. Along with sex peptides, changes in splicing of circadian clock genes can regulate responses to seasonal changes in temperature and photoperiod [44], and temperature-induced changes in day sleep are regulated by clock genes [45]. Our results suggest that there may be interesting sex-specific differences in the genetic mechanisms that underlie temperature plasticity in sleep and may permit sleep plasticity to evolve in a sex-specific manner in response to spatially varying selection.

## Conclusions

Taken together, we found novel differences in the function of sleep in a temperate and tropical population of *D. melanogaster*, where sleep increases starvation resistance in one population. We also found novel temperature plasticity responses in sleep and metabolism, some of which were sex-specific. Our study highlights the importance of studying the evolution and plasticity of sleep in the context of local adaptation. The large number of studies showing latitudinal clines for many phenotypic traits in this species [26] and the evidence that genetic differentiation in high and low latitude populations is strongly associated with clinal variation [23, 48–50] make it likely that the population differences detected here are further evidence of the myriad ways in which selection shapes latitudinal variation in this species. Nevertheless, future work on additional populations will be necessary to conclusively demonstrate that the traits studied here vary in a consistent way with latitude.

## Methods

### Fly stocks and maintenance

Experimental isofemale lines were established from Fairfield, Maine (September 2011, Latitude: 44°37′N) and Panama City, Panama (January 2012, Latitude: 8°58′N) and maintained under laboratory conditions. Ten randomly selected lines from each of the two populations were used for all experiments. Flies were maintained at a 12 h light/dark cycle on standard laboratory medium at 21 °C or 25 °C. All experimental samples were reared on standard laboratory medium at uncrowded conditions (< 75 pupae per vial), and adults were collected within the day of eclosion. Males and females were maintained together in vials containing 30–40 flies for five to 8 days until experimentation. Canton-S, Akhr^2^/CyO and Akhr^[dsRED]^/CyO lines were provided by the Thummel lab and maintained at 25 °C under uncrowded conditions.

### Behavioral analysis

Starvation resistance was measured by placing 10 females or males in a vial containing 5 mL of 1% agar dissolved in water. Number of surviving flies were measured three times a day at 21 °C and six times a day at 25 °C from six vials over at least three separate experiments. The time when 50% of the adults were dead was designated as starvation resistance.

Sleep assays were performed using Drosophila Activity Monitoring System (DAMS; TriKinetics) [51]; starvation behavioral measurements followed [19]. A total of 20–32 individuals were assayed for each genotype/sex over two separate experiments using different monitors, and at 25 °C and 21 °C. Flies were loaded into activity tubes containing 5% sucrose and 2% bactoagar (BD Difco) and acclimated for 2 days. Activity measurements for fed flies were taken from the subsequent 2 days. Incubator settings were 12 h light: 12 dark (LD) cycles at 25 °C or 21 °C. At the beginning of the daylight period on the fifth day, flies were transferred to activity tubes with 2% bactoagar to measure activity under starvation for 24 h. In these experiments, the light cycle started at 10 am local time (Zeitgeber hour 0, ZH0) and the starvation experiment was initiated at 13:00 h each day; hence the figures depicting the behavioral data for the starvation experiments start at ZH3. All genotypes and sexes were measured in each experiment to reduce environmental variability, and measurements were taken over two separate experiments for each temperature.

### Metabolic assays

Metabolic assays for whole body glucose and triglyceride were measured following Tennessen et al. (2014) and detailed descriptions are provided in Supplemental Methods. Plate-effects were accounted for using CantonS and *Akhr*^*2*^*/Akhr*^*[dsRED]*^ samples as controls. Previous studies have shown that *Akhr* mutants have elevated TGA levels and similar glucose levels as CantonS [52], and plates that significantly deviated from these patterns were removed from analysis.

### Data analysis

We used custom Perl scripts and R to analyze sleep, starvation resistance and metabolic traits. Output of statistical analyses on R are found in Supplemental File 1. All raw data files, scripts and R codes used in this manuscript are publicly available in GitHub (https://github.com/JMCridland/Sleep).

#### Sleep

To calculate sleep variables, we calculated the mean movement per minute, mean sleep bout length, number of sleep bouts and total sleep from the raw output files generated from the Drosophila Activity Monitors. These values were calculated for the entire baseline period, the entire starvation period, and the daytime and nighttime portions of these periods separately. We calculated the percentage of total sleep that occurred during the daytime and the within-fly percentage change in sleep was calculated as per Keene et al. 2010.

For the sleep data, we performed a MANOVA in R to examine the effect of temperature, sex and genotype within population on each of the sleep and movement measurements. In addition we calculated sleep values for each experiment by hour and ran a MANOVA in R to examine the effects of sex, population and hour on sleep variables. We also performed t-tests to examine differences in sleep and movement patterns between different populations, sexes and nutrition status.

#### Starvation

We calculated mean and median LD50 for each genotype. The mean and median were highly correlated (R^2^ = 0.97) and subsequent analyses were performed on the LD50 means. We performed a regression analysis and anova to examine the effects of sex, temperature and genotype within population and temperature on LD50 as well as an anova just considering sex, population and temperature.

#### Glucose and triglyceride

For glucose and triglyceride plates run at each temperature we examined the effect of plate on both the measurement divided by protein and the measurement normalized to CantonS. In general we saw smaller plate effects for Canton S normalized values.

#### LD50 and sleep

We ran an ANOVA in R to look at the effects of population, sex, temperature, percent change in sleep, and mean movement during starvation and total sleep while starved on LD50. We further performed an ANOVA to investigate the effects of sleep variables sex, population, temperature, glucose and triglyceride on LD50, as well as sex specific ANOVAs for the same variables.

As an additional analytical approach to modeling this data, we used the package brms [53, 54] in R to perform multivariate Bayesian modeling the correlation of both sleep traits and metabolic traits with starvation resistance using the same models as in the ANOVAs. This approach allows us to assign confidence intervals to our parameter estimates, examine the marginal effects of each variable on LD50, and compare posterior predictive checks to our observed data.

## Supplementary information

**Additional file 1 Supplemental Figure 1.** Starvation reduces sleep in both populations. Hourly plot of mean minutes spent sleeping and standard error over Zeitgeber Hour for (A-D) females and (E-H) males at 21 and 25 °C. Black dots indicate sleep patterns when fed, and the gray dots indicate sleep patterns under starvation. Plots start at the Zeitgeber Hour the experiment was initiated. **Supplemental Figure 2.** Regression of percent change in sleep and starvation resistance combining both populations. Regression analysis of (A) females and (B) males reared and maintained at 21 °C (blue) and 25 °C (red). Lines from ME and PC populations were combined for this analysis. R^2^ value with an asterisk denote statistical significance at *p* < 0.05. **Supplemental Figure 3.** Effect of temperature on sleep and movement. (A, C, F, H) Average sleep in minutes per Zeitgeber hour and standard error for (A) ME females, (C) PC females, (F) ME males and (H) PC males. (B, D, G, I) Average movement per 15 min per Zeitgeber hour for (B) ME females, (D) PC females, (G) ME males, and (I) PC males. (E, J) Regression plot of total percent sleep and mean movement. In all plots, red dots and lines indicate flies reared and maintained at 25 °C, and blue dots and lines indicate flies reared and maintained at 21 °C. **Supplemental Figure 4.** Summary graphics of Bayesian models investigating the relationship between starvation resistance and metabolic or behavioral parameters. (A-A’) Density plot where grey lines indicate predicted values and black line represents experimental value. (B-D) Relationship of starvation resistance and (B-B′) sex, (C-C′) temperature and (D-D’) population. Relationship of starvation resistance and (E) glucose, (F) triglyceride, (G) percent change in sleep when starved, (H) mean movement when starved and (I) total sleep when starved. Panels on the left are for the analysis using metabolic data, and panels on the right are for the analysis using behavioral parameters. **Supplemental Table 1.** Summary of starvation resistance and metabolic measurements of ME and PC flies. Mean and standard error (SE) of starvation resistance, and raw and normalized glucose and TGA levels**.** *P*-values of two-tailed t-tests are listed for each comparison, and comparisons with *p* < 0.05 are shaded in orange. **Supplemental Table 2.** Comparison of sleep and movement in ME and PC flies at 21 °C and 25 °C. Mean total percent sleep, sleep bout length in minutes, sleep bout number and mean activity for the whole day, light period, and dark period when well-fed, and whole day when starved are listed along with the standard error (SE). *P*-value for the statistical analysis comparing the two populations per sex and temperature are listed, and orange shading denotes statistical significance after Bonferroni correction. **Supplemental Table 3.** Changes in sleep and activity pattern of flies that were well-fed and starved. Mean total percent sleep, sleep bout length in minutes, sleep bout number and mean activity for the whole day for well-fed and starved flies are listed along with the standard error (SE). P-value for the statistical comparison of the fed and starved conditions are listed, and orange shading denotes statistical significance after Bonferroni correction. **Supplemental Table 4.** The effects of temperature on sleep and movement in Maine and Panama City flies. Mean total percent sleep, sleep bout length in minutes and sleep bout number for the whole day, light period, and dark period when well-fed, and whole day when starved are listed. P-value for the statistical analysis comparing flies reared at 21 °C and 25 °C are listed, and orange shading denotes statistical significance after Bonferroni correction. **Supplemental Table 5.** Linear model of starvation resistance using environmental parameters and metabolism (top) or behavior (bottom). Orange highlights denote parameters that significantly vary with starvation resistance. For the linear model that included the metabolism data, the adjusted R-squared value was 0.705 and F-statistic was 38.75 on 5 and 74 DF. For the linear model containing the sleep data, the R-squared value was 0.7795 and F-statistic was 43.02 on 6 and 73 DF. **Supplemental File 1.** Summary of R script and results for ANOVA, MANOVA, linear model and Bayesian analyses.

## Data Availability

All raw data files, scripts and R codes used in this manuscript are publicly available in GitHub (https://github.com/JMCridland/Sleep).

## Abbreviations

DAMS: Drosophila Activity Monitoring System
ME: Maine
PC: Panama City
TGA: Triglyceride
