# Supplementary material for: Phenotypic coupling of sleep and starvation resistance evolves in D. melanogaster
Source: BMC Evol Biol. 2020 Sep 22;20:126. doi: 10.1186/s12862-020-01691-8 (PMC7507639; doi:10.1186/s12862-020-01691-8)
Supplement: Supplementary file 1 — Additional file 1 Supplemental Figure 1. Starvation reduces sleep in both populations. Hourly plot of mean minutes spent sleeping and standard error over Zeitgeber Hour for (A-D) females and (E-H) males at 21 and 25 °C. Black dots indicate sleep patterns when fed, and the gray dots indicate sleep patterns under starvation. Plots start at the Zeitgeber Hour the experiment was initiated. Supplemental Figure 2. Regression of percent change in sleep and starvation resistance combining both populations. Regression analysis of (A) females and (B) males reared and maintained at 21 °C (blue) and 25 °C (red). Lines from ME and PC populations were combined for this analysis. R2 value with an asterisk denote statistical significance at p < 0.05. Supplemental Figure 3. Effect of temperature on sleep and movement. (A, C, F, H) Average sleep in minutes per Zeitgeber hour and standard error for (A) ME females, (C) PC females, (F) ME males and (H) PC males. (B, D, G, I) Average movement per 15 min per Zeitgeber hour for (B) ME females, (D) PC females, (G) ME males, and (I) PC males. (E, J) Regression plot of total percent sleep and mean movement. In all plots, red dots and lines indicate flies reared and maintained at 25 °C, and blue dots and lines indicate flies reared and maintained at 21 °C. Supplemental Figure 4. Summary graphics of Bayesian models investigating the relationship between starvation resistance and metabolic or behavioral parameters. (A-A’) Density plot where grey lines indicate predicted values and black line represents experimental value. (B-D) Relationship of starvation resistance and (B-B′) sex, (C-C′) temperature and (D-D’) population. Relationship of starvation resistance and (E) glucose, (F) triglyceride, (G) percent change in sleep when starved, (H) mean movement when starved and (I) total sleep when starved. Panels on the left are for the analysis using metabolic data, and panels on the right are for the analysis using behavioral parameters. Supplemental [file 12862_2020_1691_MOESM1_ESM.pdf]

Sarikaya et al., Phenotypic coupling of sleep and starvation resistance evolves in Drosophila melanogaster.

Supplemental materials including:

Supplemental Figure 1-4

Supplemental Table 1-5

Supplemental File 1

Supplemental Figure 1

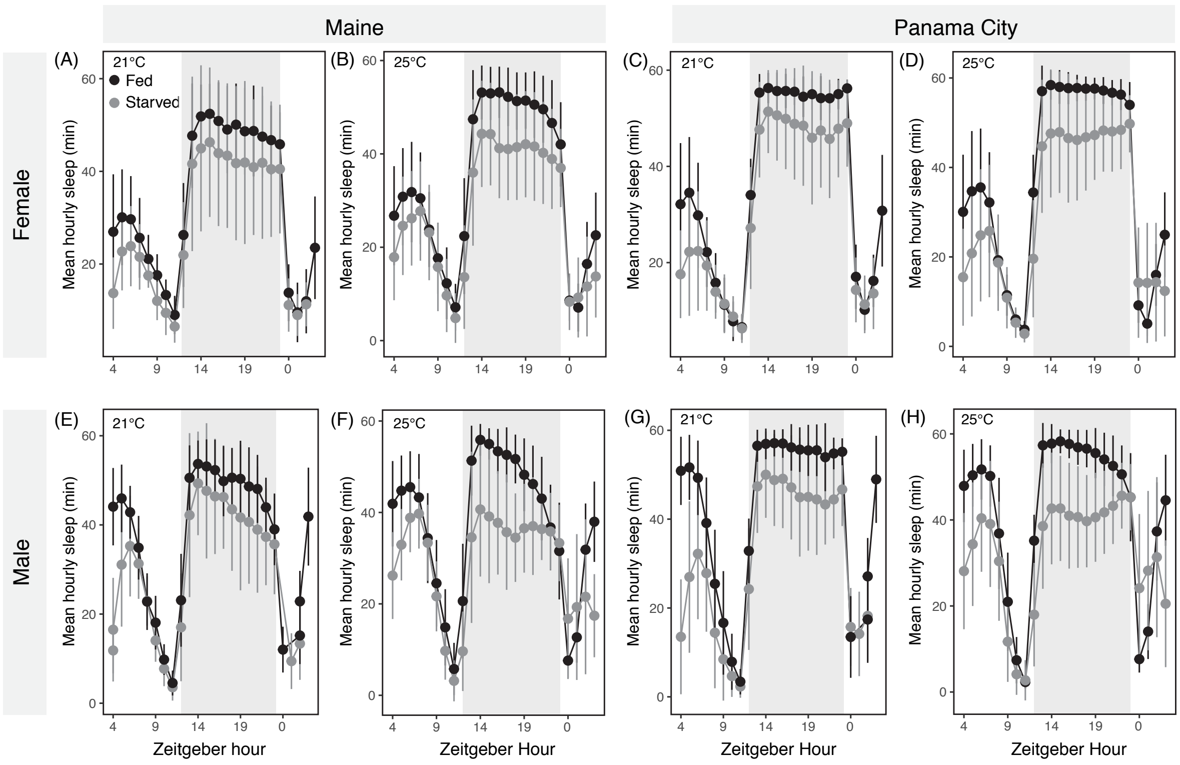

Supplemental Figure 1. Starvation reduces sleep in both populations. Hourly plot of mean minutes spent sleeping and standard error over Zeitgeber Hour for (A-D) females and (E-H) males at 21 and 25 °C. Black dots indicate sleep patterns when fed, and the gray dots indicate sleep patterns under starvation. Plots start at the Zeitgeber Hour the experiment was initiated.

Supplemental Figure 2

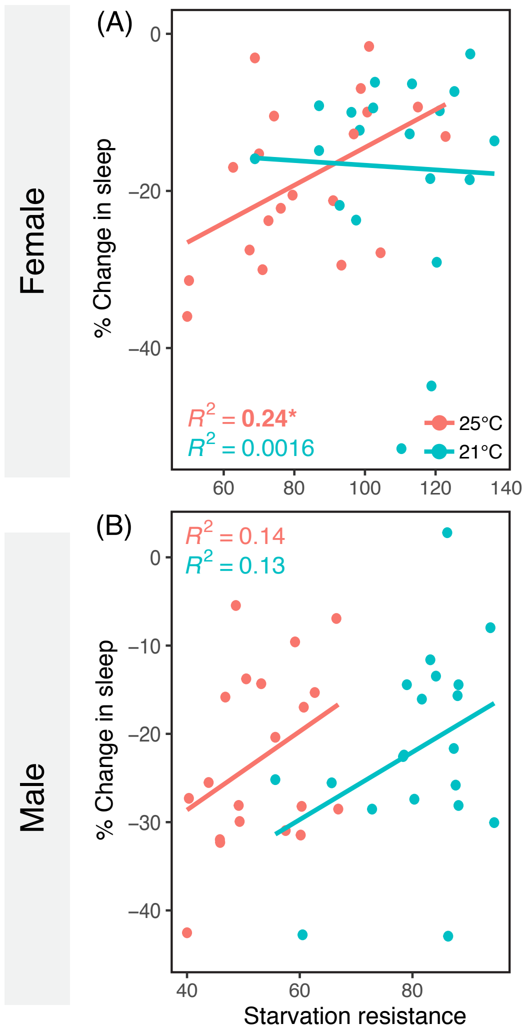

Supplemental Figure 2. Regression of percent change in sleep and starvation resistance combining both populations. Regression analysis of (A) females and (B) males reared and maintained at 21 °C (blue) and 25 °C (red). Lines from ME and PC populations were combined for this analysis. R^2 value with an asterisk denote statistical significance at p<0.05.

Supplemental Figure 3

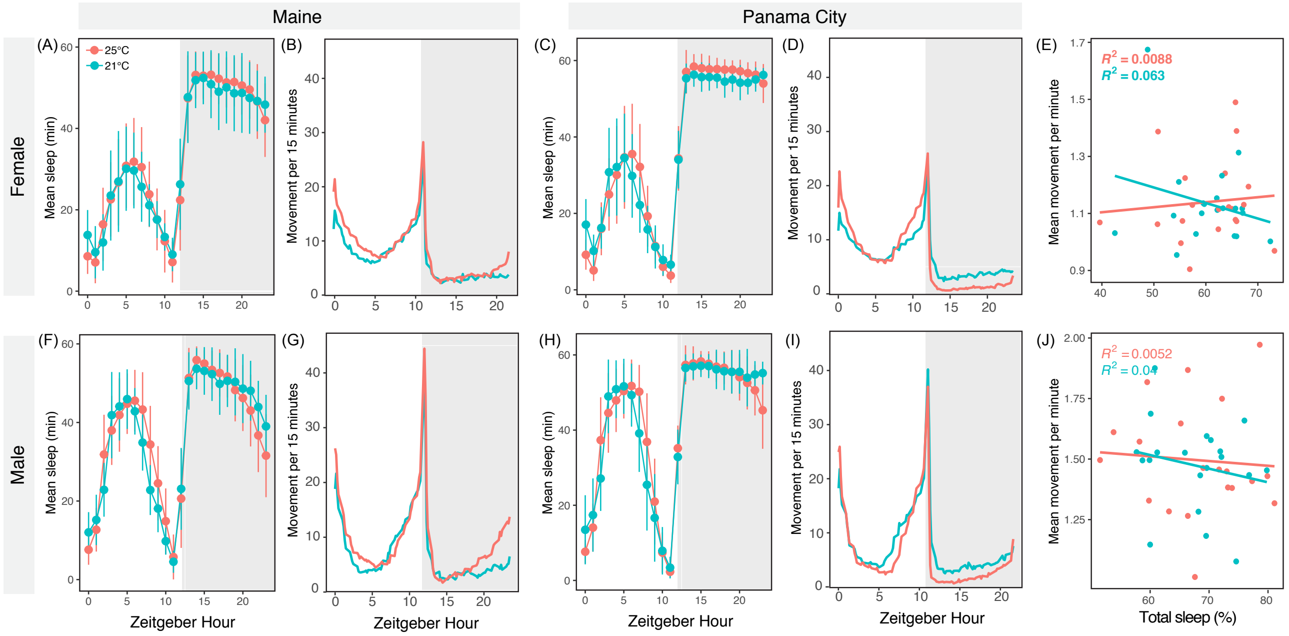

Supplemental Figure 3. Effect of temperature on sleep and movement. (A, C, F, H) Average sleep in minutes per Zeitgeber hour and standard error for (A) ME females, (C) PC females, (F) ME males and (H) PC males. (B, D, G, I) Average movement per 15 minutes per Zeitgeber hour for (B) ME females, (D) PC females, (G) ME males, and (I) PC males. (E, J) Regression plot of total percent sleep and mean movement. In all plots, red dots and lines indicate flies reared and maintained at 25 °C, and blue dots and lines indicate flies reared and maintained at 21 °C.

Supplemental Figure 4

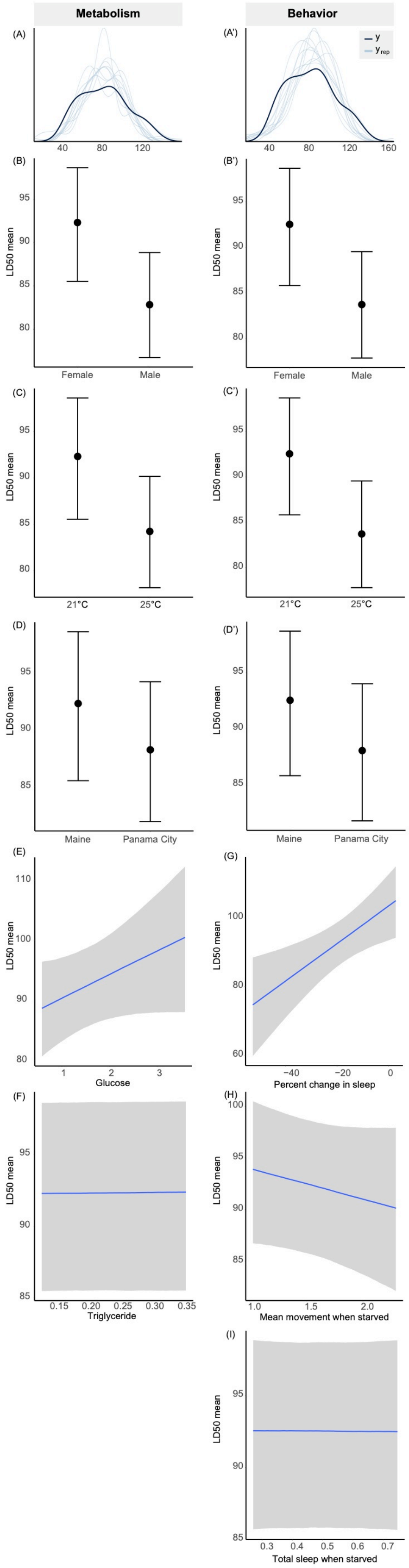

Supplemental Figure 4. Summary graphics of Bayesian models investigating the relationship between starvation resistance and metabolic or behavioral parameters. (A-A') Density plot where grey lines indicate predicted values and black line represents experimental value. (B-D) Relationship of starvation resistance and (B-B') sex, (C-C') temperature and (D-D') population. Relationship of starvation resistance and (E) glucose, (F) triglyceride, (G) percent change in sleep when starved, (H) mean movement when starved and (I) total sleep when starved. Panels on the left are for the analysis using metabolic data, and panels on the right are for the analysis using behavioral parameters.

Supplemental Table 1. Summary of starvation resistance and metabolic measurements of ME and PC flies. Mean and standard error (SE) of starvation resistance, and raw and normalized glucose and TGA levels. P-values of two-tailed t-tests are listed for each comparison, and comparisons with p<0.05 are shaded in orange.

Female

	21C							25C							
	ME			PC			ME vs PC	ME			PC			ME vs PC
	mean		SE	mean		SE	p-value	mean		SE	mean		SE	p-value
Starvation Resistance (days)	118.00	±	1.05	98.97	±	1.80	<0.0001	90.36	±	1.70	76.28	±	2.19	<0.0001
Glucose (ug Glucose/ug Protein)	68.18	±	2.40	58.37	±	1.89	0.001623	47.20	±	0.91	50.50	±	0.49	0.006
Glucose (normalized)	2.05	±	0.06	1.77	±	0.05	<0.0001	1.00	±	0.03	1.29	±	0.03	<0.0001
TGA (ug TGA/ug Protein)	232.17	±	6.69	208.46	±	5.75	<0.0001	241.41	±	5.56	216.85	±	4.64	<0.0001
TGA (normalized)	1.09	±	0.03	1.00	±	0.03	0.017							

Male

	21C							25C							
	ME			PC			ME vs PC	ME			PC			ME vs PC
	mean		SE	mean		SE	p-value	mean		SE	mean		SE	p-value
Starvation Resistance (days)	86.08	±	0.44	75.92	±	1.22	<0.0001	57.72	±	1.00	51.37	±	0.94	<0.0001
Glucose (ug Glucose/ug Protein)	45.35	±	1.65	58.15	±	1.80	0.0001046	43.80	±	0.87	50.10	±	0.59	0.000728221
Glucose (normalized)	1.43	±	0.05	1.80	±	0.21	<0.0001	1.11	±	0.03	1.40	±	0.04	<0.0001
TGA (ug TGA/ug Protein)	199.87	±	5.70	200.43	±	6.45	0.725	237.15	±	5.19	208.88	±	4.58	<0.0001
TGA (normalized)	1.18	±	0.03	1.17	±	0.04	0.401							

Supplemental Table 2. Comparison of sleep and movement in ME and PC flies at 21°C and 25°C. Mean total percent sleep, sleep bout length in minutes, sleep bout number and mean activity for the whole day, light period, and dark period when well-fed, and whole day when starved are listed along with the standard error (SE). P-value for the statistical analysis comparing the two populations per sex and temperature are listed, and orange shading denotes statistical significance after Bonferroni correction.

		21C							25C						
		ME			PC			ME vs PC	ME			PC			ME vs PC
		mean		SE	mean		SE	p-value	mean		SE	mean		SE	p-value
Total	Sleep percent (%)	57.30	±	0.72	61.96	±	0.58	<0.0001	57.51	±	0.80	63.19	±	0.58	<0.0001
	Mean bout length (min)	32.96	±	1.05	36.83	±	0.98	0.007365	36.04	±	1.61	47.64	±	1.47	<0.0001
	Mean bout number	58.67	±	1.29	56.42	±	1.25	0.2104	58.81	±	1.51	45.66	±	1.24	<0.0001
Light	Sleep percent (%)	34.30	±	0.99	34.08	±	0.96	0.8717	34.33	±	1.13	33.97	±	0.98	0.8142
	Mean bout length (min)	13.71	±	0.52	14.85	±	0.54	0.1275	15.33	±	0.65	18.13	±	0.78	0.006
	Mean bout number	19.56	±	0.50	18.24	±	0.43	0.04706	18.13	±	0.56	15.64	±	0.42	0.0004
Dark	Sleep percent (%)	80.20	±	0.94	89.72	±	0.53	<0.0001	80.59	±	0.93	92.32	±	0.48	<0.0001
	Mean bout length (min)	144.96	±	8.96	172.06	±	9.08	0.03411	121.79	±	8.39	217.86	±	12.14	<0.0001
	Mean bout number	10.03	±	0.41	10.29	±	0.41	0.6592	11.44	±	0.43	7.46	±	0.39	<0.0001
Starve total	Sleep percent (%)	46.24	±	1.01	54.45	±	0.83	<0.0001	47.47	±	1.09	51.85	±	1.09	0.005
	Mean bout length (min)	39.53	±	3.12	45.86	±	2.18	0.09723	35.45	±	1.81	50.85	±	3.64	0.0002
	Mean bout number	24.15	±	0.72	23.49	±	0.74	0.5249	25.29	±	0.77	19.63	±	0.63	<0.0001
	Percent change in sleep	-19.62	±	1.51	-11.96	±	1.18	<0.001	-17.51	±	1.65	-18.32	±	1.53	0.719
Activity (per min)	Mean total activity	1.20	±	0.02	1.11	±	0.02	0.001057	1.22	±	0.02	1.10	±	0.02	<0.0001
	Mean light activity	1.01	±	0.02	0.97	±	0.02	0.1532	1.08	±	0.02	1.02	±	0.02	0.0319
	Mean dark activity	1.94	±	0.03	2.12	±	0.04	0.0005481	1.81	±	0.03	1.85	±	0.03	0.4567
	Mean starve activity	1.34	±	0.02	1.17	±	0.01	<0.001	1.36	±	0.02	1.29	±	0.02	0.0259

Supplemental Table 3. Changes in sleep and activity pattern of flies that were well-fed and starved. Mean total percent sleep, sleep bout length in minutes, sleep bout number and mean activity for the whole day for well-fed and starved flies are listed along with the standard error (SE). P-value for the statistical comparison of the fed and starved conditions are listed, and orange shading denotes statistical significance after Bonferroni correction.

			Total Sleep (%)			Bout length (m)			Bout number			Activity		
			Fed Mean	Starved Mean	Fed vs starved p-value	Fed Mean	Starved Mean	Fed vs starved p-value	Fed Mean	Starved Mean	Fed vs starved p-value	Fed Mean	Starved Mean	Fed vs starved p-value
Female	ME	21C	57.30	46.24	<0.0001	58.67	24.15	<0.0001	32.96	39.53	0.04677	1.20	1.34	<0.0001
		25C	57.51	47.47	<0.0001	58.81	25.29	<0.0001	36.04	35.45	0.8086	1.22	1.36	<0.0001
	PC	21C	61.96	54.45	<0.0001	56.42	23.49	<0.0001	36.83	45.86	<0.0001	1.11	1.17	0.01569
		25C	63.19	51.85	<0.0001	45.66	19.63	<0.0001	47.64	50.85	0.4144	1.10	1.29	<0.0001
Male	ME	21C	63.58	50.51	<0.0001	54.47	19.72	<0.0001	38.49	46.40	0.0004766	1.61	1.69	0.03096
		25C	62.99	49.02	<0.0001	56.38	21.22	<0.0001	37.79	42.55	0.07764	1.66	1.88	<0.0001
	PC	21C	69.80	54.41	<0.0001	44.71	19.57	<0.0001	53.88	54.38	0.8574	1.44	1.50	0.05991
		25C	71.79	55.54	<0.0001	44.54	18.12	<0.0001	55.58	55.24	0.9107	1.44	1.74	<0.0001

Supplemental Table 4. The effects of temperature on sleep and movement in Maine and Panama City flies. Mean total percent sleep, sleep bout length in minutes and sleep bout number for the whole day, light period, and dark period when well-fed, and whole day when starved are listed. P-value for the statistical analysis comparing flies reared at 21 °C and 25 °C are listed, and orange shading denotes statistical significance after Bonferroni correction.

		Female						Male					
		ME			PC			ME			PC		
		21C mean	25C mean	21 vs 25C p-value	21C mean	25C mean	21 vs 25C p-value	21C mean	25C mean	21 vs 25C p-value	21C mean	25C mean	21 vs 25C p-value
Total	Total sleep (%)	57.30	57.51	0.8447	61.96	63.19	0.1357	0.64	0.63	0.5735	0.70	0.72	0.03481
	Bout length (m)	32.96	36.04	0.1108	36.83	47.64	<0.0001	38.49	37.79	0.6717	53.88	55.58	0.4922
	Bout number	58.67	58.81	0.945	56.42	45.66	<0.0001	54.47	56.38	0.24	44.71	44.54	0.9125
Light	Total sleep (%)	34.30	34.33	0.9851	34.08	33.97	0.941	0.48	0.49	0.2905	0.50	0.55	0.0006536
	Bout length (m)	13.71	15.33	0.05153	14.85	18.13	0.0005576	22.50	26.57	0.00341	31.58	38.12	0.002557
	Bout number	19.56	18.13	0.05763	18.24	15.64	<0.0001	17.30	16.87	0.4379	14.39	13.69	0.1472
Dark	Total sleep (%)	80.20	80.59	0.7644	89.72	92.32	0.0002965	0.79	0.76	0.023	0.90	0.89	0.5871
	Bout length (m)	144.96	121.79	0.05977	172.06	217.86	0.002657	128.86	90.17	<0.0001	219.30	156.95	<0.0001
	Bout number	10.03	11.44	0.01845	10.29	7.46	<0.0001	10.10	11.36	0.01592	8.28	8.74	0.3869
Starve total	Total sleep (%)	46.24	47.47	0.409	0.54	0.52	0.05811	0.51	0.49	0.2575	0.54	0.56	0.4299
	Bout length (m)	39.53	35.45	0.2581	45.86	50.85	0.2402	46.40	42.55	0.2125	54.38	55.24	0.7955
	Bout number	24.15	25.29	0.2817	23.49	19.63	<0.0001	19.72	21.22	0.06296	19.57	18.12	0.0918

Supplemental Table 5. Linear model of starvation resistance using environmental parameters and metabolism (top) or behavior (bottom). Orange highlights denote parameters that significantly vary with starvation resistance. For the linear model that included the metabolism data, the adjusted R-squared value was 0.705 and F-statistic was 38.75 on 5 and 74 DF. For the linear model containing the sleep data, the R-squared value was 0.7795 and F-statistic was 43.02 on 6 and 73 DF.

		**Estimate of B-coefficient**	**Std Error**	**p-value**
Metabolism	Sex	-27.56	3.035	<0.0001
	Temperature	-27.4236	3.6295	<0.0001
	Population	-10.081	3.1373	<0.0001
	Glucose	0.1458	3.4577	0.9664
	TGA	84.122	34.8758	0.01834
Behavior	Sex	-37.637	4.245	<0.0001
	Temperature	-28.0747	2.878	<0.0001
	Population	-14.185	3.029	<0.0001
	Percent change in sleep	-0.1999	0.2007	0.3224
	Mean mov. when starved	13.904	7.3308	0.0618
	Total sleep when starved	89.9507	23.0557	0.0002

Supplemental File 1. Summary of R script and results for ANOVA, MANOVA, linear model and Bayesian analyses.

brms_analysis_output

Julie Cridland

2020-01-14

#This section looks at the LD50 experiment.

```
    Genotype   Population Sex      Temp           ID           LD50       
 PC155  : 28   FFD:241    F:243   21C:240   F1e1   : 20   Min.   : 31.00  
 FFD29  : 25   PC :244    M:242   25C:245   F1e2   : 20   1st Qu.: 66.00  
 PC114  : 25                                F1e3   : 20   Median : 75.00  
 FFD10  : 24                                F2e1   : 20   Mean   : 81.39  
 FFD14  : 24                                F2e2   : 20   3rd Qu.: 96.00  
 FFD16  : 24                                F2e3   : 20   Max.   :165.00  
 (Other):335                                (Other):365                   
```

#Print the mean LD50 for each genotype.

```
   Genotype      LD50
1     FFD10  91.79167
2     FFD14 103.50000
3     FFD16  77.45833
4     FFD19  78.50000
5      FFD2  74.37500
6     FFD22  88.41667
7     FFD23  88.41667
8     FFD29  95.24000
9      FFD4  91.29167
10     FFD6  84.83333
11    PC113  63.91667
12    PC114  84.08000
13     PC12  97.75000
14    PC136  60.75000
15    PC141  72.66667
16    PC155  73.85714
17    PC167  73.45833
18    PC189  55.12500
19    PC200  74.78261
20     PC85  97.83333
```

#Print the standard deviation of LD50 for each genotype.

```
aggregate(LD50 ~ Genotype, data=Starve, FUN=sd)
```

```
   Genotype     LD50
1     FFD10 28.03101
2     FFD14 31.03855
3     FFD16 25.70227
4     FFD19 24.96606
5      FFD2 23.62168
6     FFD22 21.56268
7     FFD23 26.28095
8     FFD29 24.14760
9      FFD4 34.60269
10     FFD6 26.27723
11    PC113 18.40585
12    PC114 20.69001
13     PC12 30.78290
14    PC136 22.79445
15    PC141 20.07522
16    PC155 23.21193
17    PC167 14.37987
18    PC189 11.06625
19    PC200 19.97944
20     PC85 25.11827
```

#Next we perform two ANOVAs to examine the effects of variables on LD50. One analysis includes genotype as a nexted variable within population and the other does not so that we can compare the outcomes.

```
library(car)
starve_lm<-lm(LD50 ~ Sex + Temp + Population + Genotype:Population, data=Starve)
summary(starve_lm)
```

```
Call:
lm(formula = LD50 ~ Sex + Temp + Population + Genotype:Population,
    data = Starve)

Residuals:
    Min      1Q  Median      3Q     Max
-47.466  -8.253  -0.288   8.826  46.034

Coefficients: (20 not defined because of singularities)
                             Estimate Std. Error t value Pr(>|t|)
(Intercept)                 119.46567    2.94341  40.587  < 2e-16 ***
SexM                        -28.84399    1.24917 -23.091  < 2e-16 ***
Temp25C                     -26.50402    1.24990 -21.205  < 2e-16 ***
PopulationPC                  6.04167    3.97022   1.522 0.128755
PopulationFFD:GenotypeFFD14  11.70833    3.97022   2.949 0.003349 **
PopulationPC:GenotypeFFD14         NA         NA      NA       NA
PopulationFFD:GenotypeFFD16 -14.33333    3.97022  -3.610 0.000339 ***
PopulationPC:GenotypeFFD16         NA         NA      NA       NA
PopulationFFD:GenotypeFFD19 -13.29167    3.97022  -3.348 0.000881 ***
PopulationPC:GenotypeFFD19         NA         NA      NA       NA
PopulationFFD:GenotypeFFD2  -17.41667    3.97022  -4.387 1.43e-05 ***
PopulationPC:GenotypeFFD2          NA         NA      NA       NA
PopulationFFD:GenotypeFFD22  -3.37500    3.97022  -0.850 0.395721
PopulationPC:GenotypeFFD22         NA         NA      NA       NA
PopulationFFD:GenotypeFFD23  -3.37500    3.97022  -0.850 0.395721
PopulationPC:GenotypeFFD23         NA         NA      NA       NA
PopulationFFD:GenotypeFFD29   3.40153    3.93048   0.865 0.387253
PopulationPC:GenotypeFFD29         NA         NA      NA       NA
PopulationFFD:GenotypeFFD4   -0.50000    3.97022  -0.126 0.899836
PopulationPC:GenotypeFFD4          NA         NA      NA       NA
PopulationFFD:GenotypeFFD6   -6.95833    3.97022  -1.753 0.080327 .
PopulationPC:GenotypeFFD6          NA         NA      NA       NA
PopulationFFD:GenotypePC113        NA         NA      NA       NA
PopulationPC:GenotypePC113  -33.91667    3.97022  -8.543  < 2e-16 ***
PopulationFFD:GenotypePC114        NA         NA      NA       NA
PopulationPC:GenotypePC114  -12.64637    3.93048  -3.218 0.001384 **
PopulationFFD:GenotypePC12         NA         NA      NA       NA
PopulationPC:GenotypePC12    -0.08333    3.97022  -0.021 0.983263
PopulationFFD:GenotypePC136        NA         NA      NA       NA
PopulationPC:GenotypePC136  -37.08333    3.97022  -9.340  < 2e-16 ***
PopulationFFD:GenotypePC141        NA         NA      NA       NA
PopulationPC:GenotypePC141  -25.16667    3.97022  -6.339 5.50e-10 ***
PopulationFFD:GenotypePC155        NA         NA      NA       NA
PopulationPC:GenotypePC155  -22.08305    3.82684  -5.771 1.45e-08 ***
PopulationFFD:GenotypePC167        NA         NA      NA       NA
PopulationPC:GenotypePC167  -24.37500    3.97022  -6.139 1.78e-09 ***
PopulationFFD:GenotypePC189        NA         NA      NA       NA
PopulationPC:GenotypePC189  -42.70833    3.97022 -10.757  < 2e-16 ***
PopulationFFD:GenotypePC200        NA         NA      NA       NA
PopulationPC:GenotypePC200  -24.25394    4.01333  -6.043 3.11e-09 ***
PopulationFFD:GenotypePC85         NA         NA      NA       NA
PopulationPC:GenotypePC85          NA         NA      NA       NA
---
Signif. codes:  0 '***' 0.001 '**' 0.01 '*' 0.05 '.' 0.1 ' ' 1

Residual standard error: 13.75 on 463 degrees of freedom
Multiple R-squared:  0.7513,    Adjusted R-squared:   0.74
F-statistic: 66.59 on 21 and 463 DF,  p-value: < 2.2e-16
```

```
anova(starve_lm)
```

```
Analysis of Variance Table

Response: LD50
                     Df Sum Sq Mean Sq F value    Pr(>F)
Sex                   1 100559  100559 531.631 < 2.2e-16 ***
Temp                  1  85103   85103 449.917 < 2.2e-16 ***
Population            1  16764   16764  88.629 < 2.2e-16 ***
Population:Genotype  18  62094    3450  18.238 < 2.2e-16 ***
Residuals           463  87577     189
---
Signif. codes:  0 '***' 0.001 '**' 0.01 '*' 0.05 '.' 0.1 ' ' 1
```

```r
starve_lm2<-lm(LD50 ~ Sex + Temp + Population, data=Starve)
summary(starve_lm2)
```

```
Call:
lm(formula = LD50 ~ Sex + Temp + Population, data = Starve)

Residuals:
    Min      1Q  Median      3Q     Max 
-48.276 -10.854  -0.026   9.215  61.724 

Coefficients:
             Estimate Std. Error t value Pr(>|t|)    
(Intercept)   115.035      1.605   71.66  < 2e-16 ***
SexM          -28.828      1.602  -18.00  < 2e-16 ***
Temp25C       -26.422      1.602  -16.49  < 2e-16 ***
PopulationPC  -11.759      1.602   -7.34 9.17e-13 ***
---
Signif. codes:  0 '***' 0.001 '**' 0.01 '*' 0.05 '.' 0.1 ' ' 1

Residual standard error: 17.64 on 481 degrees of freedom
Multiple R-squared:  0.5749,	Adjusted R-squared:  0.5723 
F-statistic: 216.8 on 3 and 481 DF,  p-value: < 2.2e-16
```

```r
anova(starve_lm2)
```

```
Analysis of Variance Table

Response: LD50
            Df Sum Sq Mean Sq F value    Pr(>F)    
Sex          1 100559  100559 323.167 < 2.2e-16 ***
Temp         1  85103   85103 273.495 < 2.2e-16 ***
Population   1  16764   16764  53.875 9.174e-13 ***
Residuals  481 149671     311                      
---
Signif. codes:  0 '***' 0.001 '**' 0.01 '*' 0.05 '.' 0.1 ' ' 1
```

#This section examines the sleep experiment. The MANOVA looks at the effect of population, sex, temperature and genotype nested within population.

```r
sleep<-read.table("~/Dropbox/Didem_data/working/Total_sleep_all_dates_temp_edit.txt",header=TRUE)

summary(sleep)
```

```
    Date        Genotype     Pop            Num       Sex       Temp     
 May13:564   FFD14  : 118   FFD:1079   Min.   :10.0   F:1060   21C:1144  
 May18:589   FFD29  : 117   PC :1025   1st Qu.:17.0   M:1044   25C: 960  
 May3 :396   FFD10  : 115              Median :26.0                      
 May8 :555   FFD2   : 114              Mean   :25.4                      
             PC12   : 113              3rd Qu.:34.0                      
             FFD4   : 112              Max.   :41.0                      
             (Other):1415                                                
 Sleep_ALL_percent Sleep_ALL_boutNum Sleep_ALL_meanBout Sleep_ALL_meanMove
 Min.   :0.1010    Min.   :  7.00    Min.   :  7.632    Min.   :0.539     
 1st Qu.:0.5560    1st Qu.: 37.00    1st Qu.: 26.028    1st Qu.:1.064     
 Median :0.6430    Median : 50.50    Median : 35.734    Median :1.280     
 Mean   :0.6336    Mean   : 52.72    Mean   : 42.030    Mean   :1.349     
 3rd Qu.:0.7200    3rd Qu.: 66.00    3rd Qu.: 52.397    3rd Qu.:1.569     
 Max.   :0.9560    Max.   :144.00    Max.   :255.222    Max.   :4.374     
                                                                          
 Sleep_Day_percent Sleep_Day_boutNum Sleep_Day_meanBout Sleep_Day_meanMove
 Min.   :0.0050    Min.   : 1.00     Min.   :  5.00     Min.   :0.474     
 1st Qu.:0.2930    1st Qu.:11.50     1st Qu.: 11.19     1st Qu.:0.902     
 Median :0.4205    Median :16.00     Median : 16.93     Median :1.105     
 Mean   :0.4215    Mean   :16.83     Mean   : 22.25     Mean   :1.174     
 3rd Qu.:0.5550    3rd Qu.:21.00     3rd Qu.: 26.82     3rd Qu.:1.358     
 Max.   :0.9760    Max.   :48.00     Max.   :202.92     Max.   :5.170     
                                                                          
 Sleep_Night_percent Sleep_Night_boutNum Sleep_Night_meanBout
 Min.   :0.0750      Min.   : 1.00       Min.   :  7.00      
 1st Qu.:0.7827      1st Qu.: 4.50       1st Qu.: 45.65      
 Median :0.8900      Median : 8.50       Median : 90.55      
 Mean   :0.8448      Mean   : 9.75       Mean   :156.64      
```

```
 3rd Qu.:0.9460      3rd Qu.:13.50       3rd Qu.:206.05
 Max.   :0.9920      Max.   :38.00       Max.   :711.50

 Sleep_Night_meanMove Starve_ALL_percent Starve_ALL_boutNum Starve_ALL_meanBout
 Min.   :0.788        Min.   :0.0190     Min.   : 1.0       Min.   :  5.80
 1st Qu.:1.639        1st Qu.:0.4230     1st Qu.:13.0       1st Qu.: 22.45
 Median :2.020        Median :0.5335     Median :20.0       Median : 36.29
 Mean   :2.097        Mean   :0.5113     Mean   :21.5       Mean   : 46.09
 3rd Qu.:2.463        3rd Qu.:0.6240     3rd Qu.:28.0       3rd Qu.: 56.28
 Max.   :9.511        Max.   :0.9530     Max.   :82.0       Max.   :709.00

 Starve_ALL_meanMove Starve_Day_percent Starve_Day_boutNum Starve_Day_meanBout
 Min.   :0.569       Min.   :0.0070     Min.   : 1.000     Min.   :  5.000
 1st Qu.:1.166       1st Qu.:0.1678     1st Qu.: 4.000     1st Qu.:  8.493
 Median :1.411       Median :0.2940     Median : 6.000     Median : 13.608
 Mean   :1.489       Mean   :0.3033     Mean   : 6.896     Mean   : 24.282
 3rd Qu.:1.731       3rd Qu.:0.4130     3rd Qu.: 9.000     3rd Qu.: 25.186
 Max.   :4.473       Max.   :0.9450     Max.   :29.000     Max.   :252.000

 Starve_Day_meanMove Starve_Night_percent Starve_Night_boutNum
 Min.   :0.536       Min.   :0.0070       Min.   : 1.000
 1st Qu.:1.036       1st Qu.:0.5705       1st Qu.: 4.000
 Median :1.272       Median :0.8060       Median : 8.000
 Mean   :1.351       Mean   :0.7125       Mean   : 9.392
 3rd Qu.:1.565       3rd Qu.:0.9150       3rd Qu.:13.000
 Max.   :4.523       Max.   :0.9930       Max.   :45.000

 Starve_Night_meanBout Starve_Night_meanMove
 Min.   :  5.00        Min.   :0.526
 1st Qu.: 29.96        1st Qu.:1.500
 Median : 61.25        Median :1.921
 Mean   :111.57        Mean   :2.012
 3rd Qu.:133.05        3rd Qu.:2.428
 Max.   :711.00        Max.   :6.375
```

```
#a giant manova to look at everything
Allmod<-manova(cbind(Sleep_ALL_percent,Starve_ALL_percent,Sleep_Day_percent,Sleep_Night_percent,Starve_Day_percent,Starve_Night_percent,S
  ~ Pop + Sex + Temp + Pop:Genotype, data = sleep)
```

```
summary(Allmod)
```

```
               Df  Pillai approx F num Df den Df    Pr(>F)
Pop             1 0.34870   45.931     24   2059 < 2.2e-16 ***
Sex             1 0.50458   87.377     24   2059 < 2.2e-16 ***
Temp            1 0.27176   32.016     24   2059 < 2.2e-16 ***
Pop:Genotype   18 1.79746    9.596    432  37368 < 2.2e-16 ***
Residuals    2082
---
Signif. codes:  0 '***' 0.001 '**' 0.01 '*' 0.05 '.' 0.1 ' ' 1
```

```
summary.aov(Allmod)
```

```
 Response Sleep_ALL_percent :
               Df  Sum Sq Mean Sq  F value Pr(>F)
Pop             1  2.0110 2.01104 229.7823 <2e-16 ***
Sex             1  2.5705 2.57050 293.7058 <2e-16 ***
Temp            1  0.0237 0.02365   2.7023 0.1004
Pop:Genotype   18  8.1716 0.45398  51.8718 <2e-16 ***
Residuals    2082 18.2215 0.00875
---
Signif. codes:  0 '***' 0.001 '**' 0.01 '*' 0.05 '.' 0.1 ' ' 1

 Response Starve_ALL_percent :
               Df Sum Sq Mean Sq F value    Pr(>F)
Pop             1  1.736 1.73613 91.7400 < 2.2e-16 ***
Sex             1  0.280 0.28049 14.8218 0.0001217 ***
Temp            1  0.010 0.00973  0.5142 0.4734165
Pop:Genotype   18 14.067 0.78148 41.2947 < 2.2e-16 ***
Residuals    2082 39.401 0.01892
---
Signif. codes:  0 '***' 0.001 '**' 0.01 '*' 0.05 '.' 0.1 ' ' 1
```

```
 Response Sleep_Day_percent :
              Df Sum Sq Mean Sq  F value  Pr(>F)
Pop            1  0.103  0.1027   5.1092 0.02390 *
Sex            1 13.593 13.5931 676.3818 < 2e-16 ***
Temp           1  0.105  0.1048   5.2166 0.02247 *
Pop:Genotype   18 12.189  0.6772  33.6946 < 2e-16 ***
Residuals    2082 41.842  0.0201
---
Signif. codes:  0 '***' 0.001 '**' 0.01 '*' 0.05 '.' 0.1 ' ' 1

 Response Sleep_Night_percent :
              Df  Sum Sq Mean Sq  F value    Pr(>F)
Pop            1  6.3394  6.3394 714.1097 < 2.2e-16 ***
Sex            1  0.2240  0.2240  25.2337 5.511e-07 ***
Temp           1  0.0002  0.0002   0.0202    0.8869
Pop:Genotype   18 12.8205  0.7123  80.2320 < 2.2e-16 ***
Residuals    2082 18.4827  0.0089
---
Signif. codes:  0 '***' 0.001 '**' 0.01 '*' 0.05 '.' 0.1 ' ' 1

 Response Starve_Day_percent :
              Df Sum Sq Mean Sq  F value Pr(>F)
Pop            1  0.025  0.0253   1.1272 0.2885
Sex            1  4.486  4.4862 199.7934 <2e-16 ***
Temp           1  2.142  2.1425  95.4158 <2e-16 ***
Pop:Genotype   18  9.025  0.5014  22.3302 <2e-16 ***
Residuals    2082 46.750  0.0225
---
Signif. codes:  0 '***' 0.001 '**' 0.01 '*' 0.05 '.' 0.1 ' ' 1

 Response Starve_Night_percent :
              Df Sum Sq Mean Sq F value    Pr(>F)
Pop            1  5.992  5.9923 140.387 < 2.2e-16 ***
Sex            1  1.036  1.0361  24.274 9.014e-07 ***
Temp           1  2.572  2.5721  60.258 1.295e-14 ***
Pop:Genotype   18 38.984  2.1658  50.739 < 2.2e-16 ***
Residuals    2082 88.869  0.0427
---
Signif. codes:  0 '***' 0.001 '**' 0.01 '*' 0.05 '.' 0.1 ' ' 1

 Response Sleep_ALL_boutNum :
              Df Sum Sq Mean Sq F value    Pr(>F)
Pop            1  41544   41544 132.249 < 2.2e-16 ***
Sex            1  13788   13788  43.890 4.408e-11 ***
Temp           1   2439    2439   7.763  0.005381 **
Pop:Genotype   18 190854   10603  33.753 < 2.2e-16 ***
Residuals    2082 654036     314
---
Signif. codes:  0 '***' 0.001 '**' 0.01 '*' 0.05 '.' 0.1 ' ' 1

 Response Starve_ALL_boutNum :
              Df Sum Sq Mean Sq F value    Pr(>F)
Pop            1   2527  2527.1 24.7429 7.088e-07 ***
Sex            1   6668  6668.0 65.2870 1.085e-15 ***
Temp           1    196   196.4  1.9231    0.1657
Pop:Genotype   18  32480  1804.4 17.6672 < 2.2e-16 ***
Residuals    2082 212643   102.1
---
Signif. codes:  0 '***' 0.001 '**' 0.01 '*' 0.05 '.' 0.1 ' ' 1

 Response Sleep_Day_boutNum :
              Df Sum Sq Mean Sq F value    Pr(>F)
Pop            1   2977 2977.03  71.062 < 2.2e-16 ***
Sex            1   2930 2929.74  69.933 < 2.2e-16 ***
Temp           1    860  860.24  20.534  6.19e-06 ***
Pop:Genotype   18  14811  822.83  19.641 < 2.2e-16 ***
Residuals    2082  87222   41.89
---
Signif. codes:  0 '***' 0.001 '**' 0.01 '*' 0.05 '.' 0.1 ' ' 1

 Response Sleep_Night_boutNum :
              Df Sum Sq Mean Sq F value    Pr(>F)
Pop            1   2003 2002.63 63.9021 2.146e-15 ***
Sex            1     41   40.99  1.3080    0.2529
Temp           1      5    5.47  0.1746    0.6761
Pop:Genotype   18  22133 1229.59 39.2350 < 2.2e-16 ***
Residuals    2082  65248   31.34
```

```
---
Signif. codes:  0 '***' 0.001 '**' 0.01 '*' 0.05 '.' 0.1 ' ' 1

 Response Starve_Day_boutNum :
               Df  Sum Sq Mean Sq F value    Pr(>F)
Pop             1   433.0  432.97 30.2334 4.301e-08 ***
Sex             1   645.0  645.00 45.0392 2.482e-11 ***
Temp            1     1.1    1.11  0.0778    0.7803
Pop:Genotype   18  3707.0  205.95 14.3808 < 2.2e-16 ***
Residuals    2082 29816.2   14.32
---
Signif. codes:  0 '***' 0.001 '**' 0.01 '*' 0.05 '.' 0.1 ' ' 1

 Response Starve_Night_boutNum :
               Df Sum Sq Mean Sq F value    Pr(>F)
Pop             1    450  450.13  10.750 0.0010602 **
Sex             1    474  473.95  11.319 0.0007813 ***
Temp            1     83   83.12   1.985 0.1590146
Pop:Genotype   18   9347  519.27  12.401 < 2.2e-16 ***
Residuals    2082  87181   41.87
---
Signif. codes:  0 '***' 0.001 '**' 0.01 '*' 0.05 '.' 0.1 ' ' 1

 Response Sleep_ALL_meanBout :
               Df Sum Sq Mean Sq F value    Pr(>F)
Pop             1  72611   72611 185.512 < 2.2e-16 ***
Sex             1  35317   35317  90.229 < 2.2e-16 ***
Temp            1   7039    7039  17.983 2.326e-05 ***
Pop:Genotype   18 245997   13666  34.916 < 2.2e-16 ***
Residuals    2082 814920     391
---
Signif. codes:  0 '***' 0.001 '**' 0.01 '*' 0.05 '.' 0.1 ' ' 1

 Response Starve_ALL_meanBout :
               Df  Sum Sq Mean Sq F value    Pr(>F)
Pop             1   55874   55874 39.0309 5.044e-10 ***
Sex             1   24721   24721 17.2686 3.376e-05 ***
Temp            1     197     197  0.1373     0.711
Pop:Genotype   18  474961   26387 18.4324 < 2.2e-16 ***
Residuals    2082 2980462    1432
---
Signif. codes:  0 '***' 0.001 '**' 0.01 '*' 0.05 '.' 0.1 ' ' 1

 Response Sleep_Day_meanBout :
               Df Sum Sq Mean Sq F value    Pr(>F)
Pop             1  17628   17628  84.022 < 2.2e-16 ***
Sex             1 100928  100928 481.049 < 2.2e-16 ***
Temp            1   7599    7599  36.219 2.079e-09 ***
Pop:Genotype   18  72776    4043  19.271 < 2.2e-16 ***
Residuals    2082 436820     210
---
Signif. codes:  0 '***' 0.001 '**' 0.01 '*' 0.05 '.' 0.1 ' ' 1

 Response Sleep_Night_meanBout :
               Df   Sum Sq Mean Sq  F value    Pr(>F)
Pop             1  2608985 2608985 130.2973 < 2.2e-16 ***
Sex             1    70165   70165   3.5042  0.061354 .
Temp            1   197411  197411   9.8591  0.001714 **
Pop:Genotype   18  9223222  512401  25.5902 < 2.2e-16 ***
Residuals    2082 41688571   20023
---
Signif. codes:  0 '***' 0.001 '**' 0.01 '*' 0.05 '.' 0.1 ' ' 1

 Response Starve_Day_meanBout :
               Df  Sum Sq Mean Sq  F value    Pr(>F)
Pop             1   12190   12190  19.7513 9.283e-06 ***
Sex             1  167848  167848 271.9616 < 2.2e-16 ***
Temp            1   91736   91736 148.6390 < 2.2e-16 ***
Pop:Genotype   18  100641    5591   9.0593 < 2.2e-16 ***
Residuals    2082 1284960     617
---
Signif. codes:  0 '***' 0.001 '**' 0.01 '*' 0.05 '.' 0.1 ' ' 1

 Response Starve_Night_meanBout :
               Df   Sum Sq Mean Sq F value    Pr(>F)
Pop             1  1016445 1016445  60.476 1.162e-14 ***
Sex             1   287856  287856  17.127 3.636e-05 ***
```

```
Temp            1   503453  503453  29.954 4.956e-08 ***
Pop:Genotype   18  4370633  242813  14.447 < 2.2e-16 ***
Residuals    2082 34992827   16807
---
Signif. codes:  0 '***' 0.001 '**' 0.01 '*' 0.05 '.' 0.1 ' ' 1

 Response Sleep_ALL_meanMove :
               Df  Sum Sq Mean Sq  F value Pr(>F)
Pop             1  12.152  12.152 107.4913 <2e-16 ***
Sex             1  76.039  76.039 672.6020 <2e-16 ***
Temp            1   0.149   0.149   1.3186  0.251
Pop:Genotype   18  28.847   1.603  14.1757 <2e-16 ***
Residuals    2082 235.374   0.113
---
Signif. codes:  0 '***' 0.001 '**' 0.01 '*' 0.05 '.' 0.1 ' ' 1

 Response Starve_ALL_meanMove :
               Df  Sum Sq Mean Sq F value    Pr(>F)
Pop             1  12.932  12.932  90.718 < 2.2e-16 ***
Sex             1  83.543  83.543 586.077 < 2.2e-16 ***
Temp            1  10.715  10.715  75.167 < 2.2e-16 ***
Pop:Genotype   18  35.301   1.961  13.758 < 2.2e-16 ***
Residuals    2082 296.783   0.143
---
Signif. codes:  0 '***' 0.001 '**' 0.01 '*' 0.05 '.' 0.1 ' ' 1

 Response Sleep_Day_meanMove :
               Df  Sum Sq Mean Sq F value    Pr(>F)
Pop             1   2.904   2.904  26.435  2.98e-07 ***
Sex             1  52.231  52.231 475.514 < 2.2e-16 ***
Temp            1   1.627   1.627  14.810 0.0001225 ***
Pop:Genotype   18  27.070   1.504  13.692 < 2.2e-16 ***
Residuals    2082 228.687   0.110
---
Signif. codes:  0 '***' 0.001 '**' 0.01 '*' 0.05 '.' 0.1 ' ' 1

 Response Sleep_Night_meanMove :
               Df Sum Sq Mean Sq  F value    Pr(>F)
Pop             1   0.62   0.625   1.7707    0.1834
Sex             1  55.78  55.785 158.0484 < 2.2e-16 ***
Temp            1  14.08  14.076  39.8789 3.294e-10 ***
Pop:Genotype   18  94.35   5.242  14.8504 < 2.2e-16 ***
Residuals    2082 734.86   0.353
---
Signif. codes:  0 '***' 0.001 '**' 0.01 '*' 0.05 '.' 0.1 ' ' 1

 Response Starve_Day_meanMove :
               Df  Sum Sq Mean Sq F value    Pr(>F)
Pop             1   5.019   5.019  35.369 3.192e-09 ***
Sex             1  57.830  57.830 407.541 < 2.2e-16 ***
Temp            1  15.237  15.237 107.378 < 2.2e-16 ***
Pop:Genotype   18  33.689   1.872  13.190 < 2.2e-16 ***
Residuals    2082 295.436   0.142
---
Signif. codes:  0 '***' 0.001 '**' 0.01 '*' 0.05 '.' 0.1 ' ' 1

 Response Starve_Night_meanMove :
               Df Sum Sq Mean Sq  F value    Pr(>F)
Pop             1   2.10   2.100   4.8871   0.02717 *
Sex             1  85.17  85.169 198.2452 < 2.2e-16 ***
Temp            1  11.42  11.419  26.5792 2.769e-07 ***
Pop:Genotype   18 108.75   6.041  14.0626 < 2.2e-16 ***
Residuals    2082 894.45   0.430
---
Signif. codes:  0 '***' 0.001 '**' 0.01 '*' 0.05 '.' 0.1 ' ' 1
```

#This section combines the sleep experiment data with the LD50 data.

```
master<-read.table("~/Dropbox/Didem_data/working/Master_table_new_temp_edit.txt",header=TRUE)

summary(master)
```

```
    Genotype   Pop     Sex     Temp      LD50_mean        LenF_mean
 FFD10   : 4   FFD:40   F:40   21C:40   Min.   : 40.00   Min.   :1.219
 FFD14   : 4   PC :40   M:40   25C:40   1st Qu.: 60.66   1st Qu.:1.369
 FFD16   : 4                            Median : 81.00   Median :1.475
```

```
 FFD19   : 4                              Mean   : 81.49   Mean   :1.472  
 FFD2    : 4                              3rd Qu.: 97.75   3rd Qu.:1.548  
 FFD22   : 4                              Max.   :136.67   Max.   :1.771  
 (Other):56                                                NA's   :1      
   LenQ_mean       PerChange          GluNorm           GluCanS     
 Min.   :0.960   Min.   :-52.807   Min.   :0.02900   Min.   :0.583  
 1st Qu.:1.264   1st Qu.:-28.108   1st Qu.:0.04350   1st Qu.:1.140  
 Median :1.369   Median :-18.498   Median :0.05000   Median :1.357  
 Mean   :1.355   Mean   :-19.976   Mean   :0.05262   Mean   :1.478  
 3rd Qu.:1.432   3rd Qu.:-12.107   3rd Qu.:0.05600   3rd Qu.:1.693  
 Max.   :1.647   Max.   :  2.792   Max.   :0.12800   Max.   :3.572  
 NA's   :1                                                          
    TGANorm          TGACanS      Sleep_ALL_percent Sleep_ALL_meanMove
 Min.   :0.1130   Min.   :0.780   Min.   :0.4060    Min.   :0.929     
 1st Qu.:0.1857   1st Qu.:0.985   1st Qu.:0.5835    1st Qu.:1.138     
 Median :0.2100   Median :1.101   Median :0.6370    Median :1.323     
 Mean   :0.2184   Mean   :1.108   Mean   :0.6323    Mean   :1.347     
 3rd Qu.:0.2415   3rd Qu.:1.244   3rd Qu.:0.6817    3rd Qu.:1.550     
 Max.   :0.3390   Max.   :1.582   Max.   :0.8050    Max.   :2.032     
                  NA's   :40                                          
 Sleep_Day_percent Sleep_Day_meanMove Sleep_Night_percent Sleep_Night_meanMove
 Min.   :0.1630    Min.   :0.795      Min.   :0.5420      Min.   :1.501       
 1st Qu.:0.3520    1st Qu.:1.002      1st Qu.:0.7708      1st Qu.:1.844       
 Median :0.4175    Median :1.145      Median :0.8825      Median :2.075       
 Mean   :0.4214    Mean   :1.172      Mean   :0.8422      Mean   :2.080       
 3rd Qu.:0.5060    3rd Qu.:1.335      3rd Qu.:0.9247      3rd Qu.:2.319       
 Max.   :0.6830    Max.   :1.803      Max.   :0.9590      Max.   :2.962       
                                                                             
 Starve_ALL_percent Starve_ALL_meanMove Starve_Day_percent Starve_Day_meanMove
 Min.   :0.2700     Min.   :1.028       Min.   :0.1390     Min.   :0.901      
 1st Qu.:0.4400     1st Qu.:1.265       1st Qu.:0.2417     1st Qu.:1.175      
 Median :0.5105     Median :1.417       Median :0.2820     Median :1.290      
 Mean   :0.5051     Mean   :1.493       Mean   :0.3066     Mean   :1.353      
 3rd Qu.:0.5703     3rd Qu.:1.707       3rd Qu.:0.3922     3rd Qu.:1.537      
 Max.   :0.7480     Max.   :2.293       Max.   :0.5800     Max.   :2.168      
                                                                             
 Starve_Night_percent Starve_Night_meanMove
 Min.   :0.2840       Min.   :1.264        
 1st Qu.:0.5595       1st Qu.:1.697        
 Median :0.7365       Median :1.988        
 Mean   :0.6974       Mean   :1.989        
 3rd Qu.:0.8325       3rd Qu.:2.276        
 Max.   :0.9520       Max.   :2.738        
```

```
Mmod<-lm(LD50_mean ~ Sex + Temp + Pop + PerChange + Starve_ALL_meanMove + Starve_ALL_percent, data =
  master)

print("QR rank for model")
```

```
[1] "QR rank for model"
```

```
qr(Mmod)$rank
```

```
[1] 7
```

```
summary(Mmod)
```

```

Call:
lm(formula = LD50_mean ~ Sex + Temp + Pop + PerChange + Starve_ALL_meanMove + 
    Starve_ALL_percent, data = master)

Residuals:
     Min       1Q   Median       3Q      Max 
-26.0780  -6.5568  -0.1559   7.3909  30.3844 

Coefficients:
                    Estimate Std. Error t value Pr(>|t|)    
(Intercept)          51.2540    17.4901   2.930 0.004515 ** 
SexM                -37.6376     4.2451  -8.866 3.32e-13 ***
Temp25C             -28.0747     2.8780  -9.755 7.25e-15 ***
PopPC               -14.1851     3.0296  -4.682 1.28e-05 ***
```

```
PerChange            -0.1999     0.2007  -0.996 0.322468
Starve_ALL_meanMove  13.9041     7.3308   1.897 0.061829 .
Starve_ALL_percent   89.9507    23.0557   3.901 0.000211 ***
---
Signif. codes:  0 '***' 0.001 '**' 0.01 '*' 0.05 '.' 0.1 ' ' 1

Residual standard error: 12 on 73 degrees of freedom
Multiple R-squared:  0.7795,    Adjusted R-squared:  0.7614
F-statistic: 43.02 on 6 and 73 DF,  p-value: < 2.2e-16
```

```
summary.aov(Mmod)
```

```
                    Df Sum Sq Mean Sq F value   Pr(>F)
Sex                  1  16616   16616 115.381  < 2e-16 ***
Temp                 1  14052   14052  97.575 4.29e-15 ***
Pop                  1   2743    2743  19.048 4.13e-05 ***
PerChange            1   1250    1250   8.683 0.004309 **
Starve_ALL_meanMove  1    321     321   2.229 0.139730
Starve_ALL_percent   1   2192    2192  15.221 0.000211 ***
Residuals           73  10513     144
---
Signif. codes:  0 '***' 0.001 '**' 0.01 '*' 0.05 '.' 0.1 ' ' 1
```

```
Metmod<-lm(LD50_mean ~ Sex + Temp + Pop + GluCanS + TGANorm, data=master)

print("QR rank for model")
```

```
[1] "QR rank for model"
```

```
qr(Metmod)$rank
```

```
[1] 6
```

```
summary(Metmod)
```

```
Call:
lm(formula = LD50_mean ~ Sex + Temp + Pop + GluCanS + TGANorm,
    data = master)

Residuals:
    Min      1Q  Median      3Q     Max
-32.414  -8.761  -0.833   8.913  37.489

Coefficients:
            Estimate Std. Error t value Pr(>|t|)
(Intercept)  95.4367     9.3641  10.192 9.69e-16 ***
SexM        -27.5623     3.0351  -9.081 1.17e-13 ***
Temp25C     -27.4239     3.6295  -7.556 8.93e-11 ***
PopPC       -10.0881     3.1373  -3.216  0.00193 **
GluCanS       0.1458     3.4577   0.042  0.96648
TGANorm      84.1220    34.8758   2.412  0.01834 *
---
Signif. codes:  0 '***' 0.001 '**' 0.01 '*' 0.05 '.' 0.1 ' ' 1

Residual standard error: 13.35 on 74 degrees of freedom
Multiple R-squared:  0.7236,    Adjusted R-squared:  0.705
F-statistic: 38.75 on 5 and 74 DF,  p-value: < 2.2e-16
```

```
summary.aov(Metmod)
```

```
            Df Sum Sq Mean Sq F value   Pr(>F)
Sex          1  16616   16616  93.301 9.56e-15 ***
Temp         1  14052   14052  78.903 2.77e-13 ***
Pop          1   2743    2743  15.403 0.000193 ***
GluCanS      1     61      61   0.345 0.558672
TGANorm      1   1036    1036   5.818 0.018343 *
Residuals   74  13179     178
```

```
---
Signif. codes:  0 '***' 0.001 '**' 0.01 '*' 0.05 '.' 0.1 ' ' 1
```

#This section describes the brms anaysis for LD50 and sleep data.

```
master_model<-brms::brm(LD50_mean ~ Sex + Temp + Pop + PerChange + Starve_ALL_meanMove +
  Starve_ALL_percent, data = master, family = 'gaussian',
  prior=set_prior('normal(0,3)'),iter=10000,chains = 4, cores = 8,control=list(adapt_delta =
  0.99999,max_treedepth=15))
```

```
summary(master_model)
```

```
 Family: gaussian
  Links: mu = identity; sigma = identity
Formula: LD50_mean ~ Sex + Temp + Pop + PerChange + Starve_ALL_meanMove + Starve_ALL_percent
   Data: master (Number of observations: 80)
Samples: 4 chains, each with iter = 10000; warmup = 5000; thin = 1;
         total post-warmup samples = 20000

Population-Level Effects:
                    Estimate Est.Error l-95% CI u-95% CI Rhat Bulk_ESS Tail_ESS
Intercept             107.79      6.47    94.88   120.26 1.00    18548    13805
SexM                   -8.71      2.73   -14.03    -3.35 1.00    15895    14454
Temp25C                -8.80      2.63   -13.80    -3.50 1.00    15473    14332
PopPC                  -4.46      2.51    -9.32     0.47 1.00    17724    13713
PerChange               0.54      0.19     0.17     0.93 1.00    17160    12773
Starve_ALL_meanMove    -2.98      2.83    -8.49     2.54 1.00    18214    14439
Starve_ALL_percent     -0.02      2.98    -5.77     5.83 1.00    18945    14286

Family Specific Parameters:
      Estimate Est.Error l-95% CI u-95% CI Rhat Bulk_ESS Tail_ESS
sigma    18.56      1.85    15.29    22.52 1.00    12480    13878

Samples were drawn using sampling(NUTS). For each parameter, Eff.Sample
is a crude measure of effective sample size, and Rhat is the potential
scale reduction factor on split chains (at convergence, Rhat = 1).
```

```
print(marginal_effects(master_model), ask=FALSE)
```

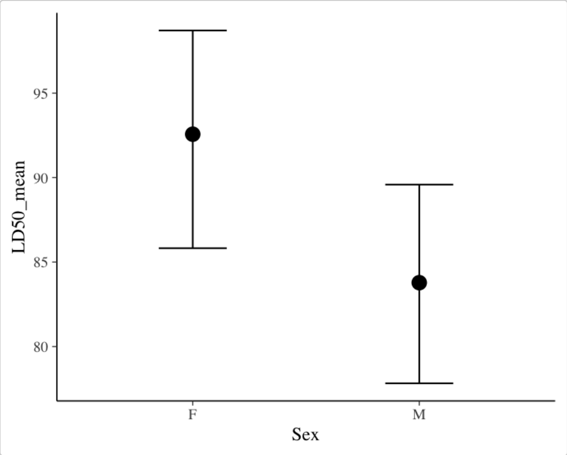

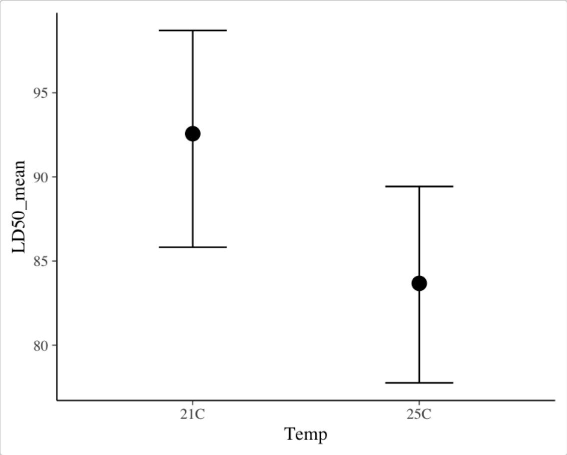

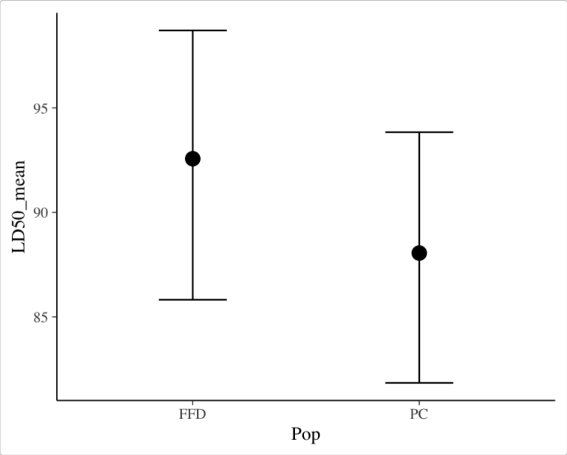

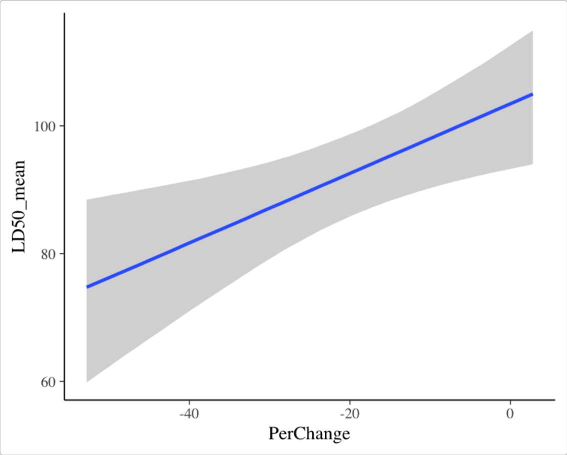

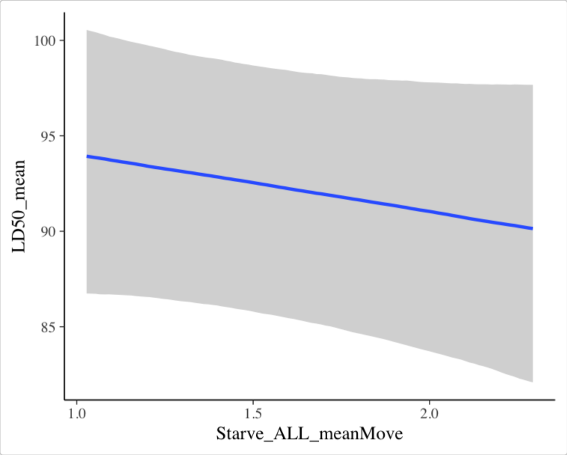

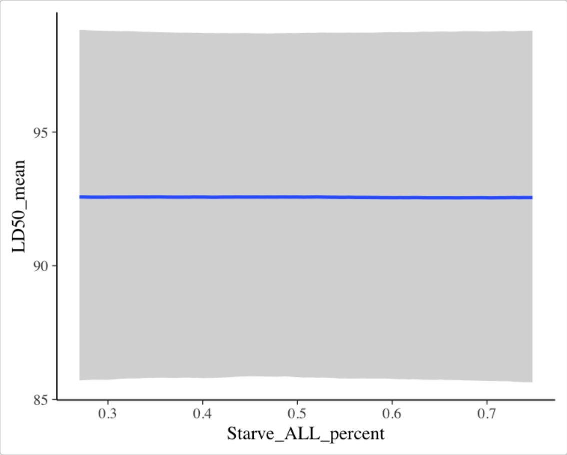


```
ppmaster=brms::pp_check(master_model)
ppmaster
```

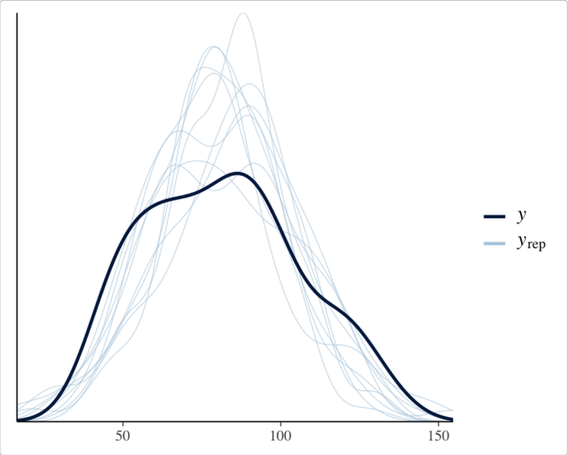

#This section describes the brms analysis for LD50 and metabolism data.

```
metabolism_model<-brms::brm(LD50_mean ~ Sex + Temp + Pop + GluCanS + TGANorm, data=master, family =
  'gaussian', prior=set_prior('normal(0,3)'),iter=10000,chains = 4, cores = 8,control=list(adapt_delta =
  0.99999,max_treedepth=15))
```

```
summary(metabolism_model)
```

```
 Family: gaussian
  Links: mu = identity; sigma = identity
Formula: LD50_mean ~ Sex + Temp + Pop + GluCanS + TGANorm
   Data: master (Number of observations: 80)
Samples: 4 chains, each with iter = 10000; warmup = 5000; thin = 1;
         total post-warmup samples = 20000

Population-Level Effects:
          Estimate Est.Error l-95% CI u-95% CI Rhat Bulk_ESS Tail_ESS
Intercept    86.27      4.99    76.49    96.05 1.00    17297    14549
SexM         -9.38      2.74   -14.71    -3.92 1.00    15537    13823
Temp25C      -7.99      2.70   -13.23    -2.68 1.00    16422    13364
PopPC        -4.06      2.53    -8.95     0.95 1.00    17100    13705
GluCanS       3.95      2.46    -0.92     8.79 1.00    17895    14367
TGANorm       0.42      2.97    -5.38     6.20 1.00    17814    14044

Family Specific Parameters:
      Estimate Est.Error l-95% CI u-95% CI Rhat Bulk_ESS Tail_ESS
sigma    19.27      1.91    15.86    23.37 1.00    13688    14638

Samples were drawn using sampling(NUTS). For each parameter, Eff.Sample
is a crude measure of effective sample size, and Rhat is the potential
scale reduction factor on split chains (at convergence, Rhat = 1).
```

```
print(marginal_effects(metabolism_model), ask=FALSE)
```

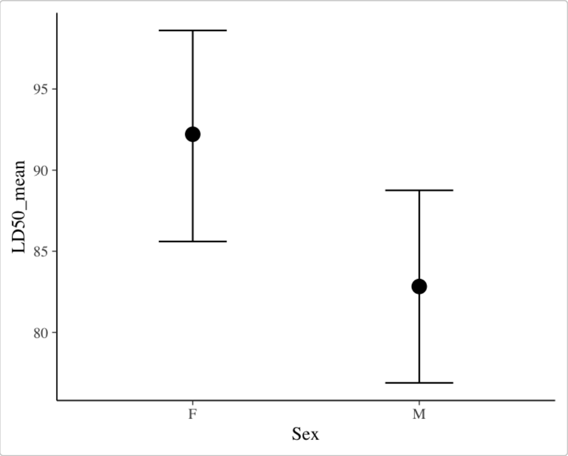

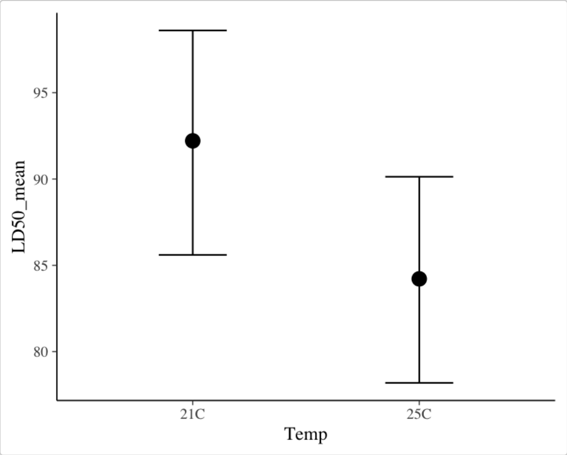

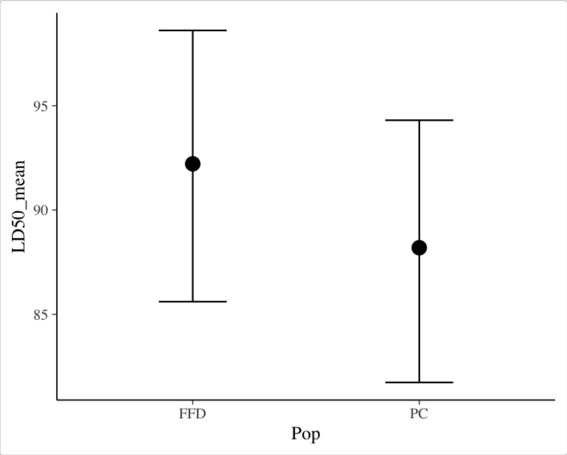

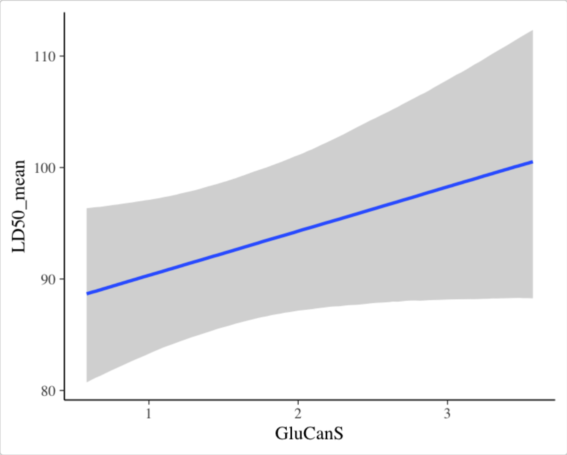

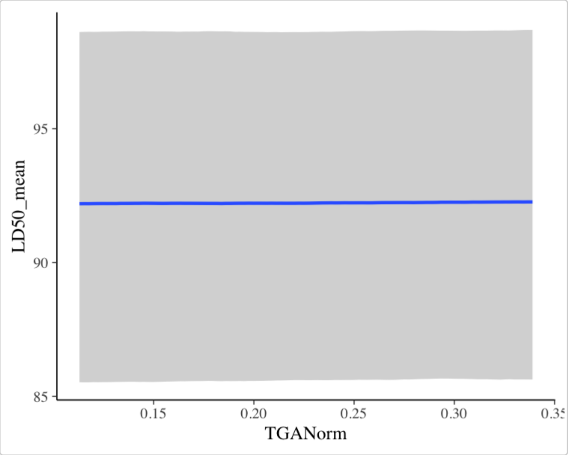


```
ppmet=brms::pp_check(metabolism_model)
ppmet
```

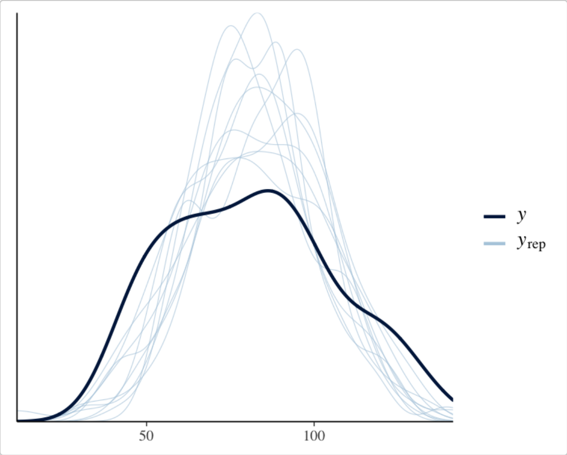
50
100
y
y_rep
